# Supplementary material for: Forecasting of phenotypic and genetic outcomes of experimental evolution in Pseudomonas protegens
Source: PLoS Genet. 2021 Aug 5;17(8):e1009722. doi: 10.1371/journal.pgen.1009722 (PMC8370652; doi:10.1371/journal.pgen.1009722)
Supplement: S1 Text — (DOCX) [file pgen.1009722.s008.docx]

**S1 Text**

Pentz and Lind

*Forecasting of phenotypic and genetic outcomes of experimental evolution in Pseudomonas protegens*

**Forecasting experimental evolution of *P. protegens* Pf-5**

All evolutionary forecasts are dependent on previous knowledge to some degree, in terms of theory and empirical data, combined with an assessment of how likely these are to be applicable to the specific novel situation. Therefore, it is necessary to describe the bases for predictions on different biological levels together with possible alternative outcomes to be able to judge the underlying assumptions and potential biases influencing the forecasts and to determine why predictions fail.

Forecasting experimental evolution in a novel context (*e.g.*, a new species or different environment) first requires a prediction of the dominant selective pressures and the corresponding phenotypes that will have the highest fitness. Only then can more specific phenotypic predictions be made, followed by genetic predictions when the genotype-to-phenotype map is well characterized. Thus, predictions of specific phenotypes are dependent on a correct prediction of dominant selective pressures and any genetic predictions will be dependent on correct phenotypic predictions. Therefore, we describe predictions on different biological levels in the order of phenotypes, genetics and molecular details.

**Prediction 1: Mutants with increased ability to colonize the air-liquid interface by increased cell-cell or cell-wall adhesion will evolve and rise to high frequencies as these will be among the most fit classes of single step adaptive mutants**

It is likely that there are several evolutionary strategies with increased fitness under the experimental conditions used here, but we predict that selection for access to oxygen will be the dominant selective pressure. Mutants with an increased ability to colonize the air-liquid interface will be one of the fittest class of adaptive mutants and rapidly increase in frequency due to the large increase in growth rate under high oxygen conditions (Fig 1A). This is likely to be true only when the wild type is a poor colonizer of the air-liquid interface. The cell wall of Gram-negative bacteria, such as *Pseudomonas*, has several components that may be used to increase cell-to-cell adhesion and attachment to surfaces to allow colonization of the air-liquid interface (Fig 1B). Thus, all bacteria that are obligate aerobes, like Pf-5, are predicted to be able to mutate to find a phenotypic solution to colonize the air-liquid interface under conditions where selection for access to oxygen is strong.

**Prediction 2: Mutants that use exopolysaccharides will have higher fitness than alternative phenotypic solutions with increased air-liquid interface colonization, such as lipopolysaccharides, capsular polysaccharides, cell-chaining or adhesive proteins (Fig 1B).** As Pf-5 does not have genes required for biosynthesis of cellulose it is predicted to instead use Pel exopolysaccharide. If mutants specializing in colonizing the air-liquid interface are the among the fittest classes of single step adaptive mutants, we can make a prediction of the specific phenotypic solutions used for increasing cell-to-cell and/or cell-surface adhesion. A key parameter here is the stability of the mat, as it will collapse and fall to the bottom when a self-supporting mat covering the surface cannot be maintained. In addition to different exopolysaccharides (EPSs), capsular polysaccharides (CPSs) and lipopolysaccharides (LPS) might also be used (Fig 1B). Adhesive proteins and incomplete cleavage of the peptidoglycan (PG) layer are also potential phenotypic solutions for increased cell-cell adhesion. In SBW25, at least four alternative distinct phenotypes with divergent colony morphology are selected for at the surface, involving an alternative EPS, CPS, LPS and PG, but they all have lower fitness than the WS type that form cellulose-based mats [1–4]. Pf-5 lacks genes for cellulose biosynthesis, but there are other EPSs that might be used (*e.g.*, Pel, Psl, polyglucosamine (PGA)). Thus, we predict that overexpression of an EPS will always provide the most stable mats and therefore have the highest fitness (Fig 1D) and that Pel will be the primary EPS used based on its importance for pellicle formation at the air-liquid interface in *Pseudomonas aeruginosa* PA14 [5], the lack of mutants using Psl in SBW25, and the lower fitness of mutants using PGA in SBW25 [2]. This prediction might fail, though, because the encoded EPS does not form superior mats, is non-functional, or the biosynthetic costs of EPS production outweighs its benefit relative to other phenotypic solutions.

**Prediction 3: The most fit mutants will have reduced motility due to activation of DGCs resulting in increased c-di-GMP and exopolysaccharide production and selection against the cost of motility in biofilms**

In SBW25 and *Pseudomonas aeruginosa* PA14, overexpression of EPSs used for mat formation are linked to mutations increasing c-di-GMP production rather than mutations in the promoters of, or genes in, the EPS operons themselves [2,6,7]. This can be explained by the role of post-translation regulation by c-di-GMP in the production of the EPSs cellulose, Pel, PGA and alginate [8]. Furthermore, there may be an additional benefit of using c-di-GMP activation in that it reduces motility, which is not needed when bacteria are established at the air-liquid interface and may be antagonistic to biofilm formation [9]. Motility also consumes a large amount of energy and thus is likely to be lost when not under selection [10,11]. Consequently, we also predict that the fittest mutants overexpressing EPSs will have reduced motility (Fig 1D).

**Prediction 4: Most mutations will cause loss of function, followed by promoter mutations and even less frequent activating mutations and double inactivating mutations.**

**Prediction 5: Mutations in the molecular networks of the negatively regulated DGCs WspR, AwsR and MwsR will be the most common route to WS due to a large mutational target size**

Predictions on the genetic level are more difficult depending on the greater dimensionality of the problem (*i.e.*, mutations in many genes can lead to the same phenotype) but also because genetic predictions will be conditional on a correct phenotypic prediction. The genotype-to-phenotype map must also be conserved to some degree between species, so that similar mutations give rise to similar, previously observed, phenotypes. Mutational biases can also greatly skew the diversity of mutants found for a particular species, which can cause predictions to fail even if the theoretical model of the genotype-to-phenotype map is accurate.

In cases where the phenotypic prediction fails, for example because a novel previously unknown phenotypic solution to the adaptive problem is used, or the genotype-to-phenotype map is not conserved, detailed genetic predictions will also inevitably fail. However, a general prediction of the types of mutations is still possible. Loss-of-function mutations account for a majority of adaptive mutations in many experimental evolution studies [12], presumably because of a large mutational target size. This is true also for SBW25 where a large majority of WS mutations are loss-of-function mutations in negative regulators (WspF, AwsX) or intragenic negative regulatory regions (MwsR, PFLU0085) of the main DGCs [13] (Fig 1D) as well as loss-of-function mutations in the genes underpinning the alternative phenotypes [1,2,13]. When the negatively regulated pathways to WS are removed, DGCs are instead activated by promoter mutations, including promoter captures followed by more rare intragenic activating mutations [6] (Fig 1C).

**Prediction 6: A previously developed mathematical model predicts that most mutations will be in WspA, WspE, WspF, AwsR, AwsX and MwsR.**

If WS types in Pf-5 evolve using mutational activation of DGCs, as expected if the genotype-to-phenotype map is conserved, we can use previously developed mathematical models [14] to predict the relative rates for the three main pathways (Wsp, Aws, and Mws) to the WS phenotype based on a detailed understanding of the molecular functions of the proteins involved (Fig 1E, S1 Text Methods). The molecular networks of the three main pathways (Wsp, Aws, and Mws) were modeled as a system of ordinary differential equations (S1 Text Methods, S1 Fig, S2 Fig) and this work is fully described as Model IV in Lind et al. 2019 [14]. The functional interactions between components of the networks, for example an enzymatic reaction or a conformational change in a protein, are described by biochemical reaction rates that can be either increased or decreased by mutations, resulting in changes in the concentration of the active form of the DGC of each pathway. We assume here that the functions of the proteins in the Wsp, Aws, and Mws regulatory networks are conserved between SBW25 and Pf-5. We do not assume that the concentrations of the proteins or the biochemical reaction rates are identical as these are unknown for both species and are repeatedly drawn from uniform distributions. This allows us to estimate, by numerical simulations, the relative probability that mutational changes in reaction rates for each pathway result in a phenotypic change in the form of a wrinkly spreader. The model is independent of the exopolysaccharide used as the structural component, as it only calculates the probability that a mutation increases the active form of the DGC for each pathway. The model can also incorporate information about mutational biases by increasing probability of changes in specific reaction rates, which improved the predictive ability of the model in Lind et al. 2019 [14]. We do not assume that mutational hot spot sites are conserved and therefore all reaction rates had the same probability of mutational changes. Based on data from SBW25 [14] and previous data on effects of random mutations [15] and the general expectation that disabling mutations (reducing reaction rates) are more likely than enabling mutations (increasing reaction rates), we assumed that disabling changes are ten times more common (Fig 1E).

The mathematical model of the main DGC pathways can also predict the relative contribution of changes to each reaction rate to phenotypic change ([14], S1 Text Methods). With an understanding of the functions of the proteins involved this allows prediction of functional effects of mutations and thereby also targeted regions in the proteins. Here we make the simplifying assumption that changes to a reaction rate is equally likely to be caused by mutations in all participating proteins. With more data on mutational target size of each protein it would be possible to integrate this information, which might improve future predictions. High rates of WS mutations are predicted for WspF, WspA, WspE, AwsX and AwsR and MwsR (Fig 1E). A significantly lower rate of enabling mutations is also predicted to occur in WspC, WspR and AwsO (Fig 1E)*.* Despite the simplicity of the null model, it closely predicted the mutational targets in SBW25 with equal rates for WspF, WspA and WspE and rare mutations in WspC and WspR, suggesting that it is a useful null model for other species [14].

**Prediction 7: Mutated regions for proteins in the Wsp, Aws and Mws pathways are predicted based on previous mutations in SBW25 and *P. aeruginosa* combined with analysis of homology models of protein structures (Fig 2).**

If the genotype-to-phenotype map is conserved between SBW25 and Pf-5 and modeling allows prediction of mutated genes and their functional effects on reaction rates, we can proceed to make more detailed predictions about the specific mutations for each gene. The level of parallelism at the nucleotide level between species is expected to be dependent on the number of possible mutations to WS and the degree of functional conservation of the proteins involved that define the genotype-to-phenotype map. For Pf-5, the genes in the four main pathways (Wsp, Aws, Mws, PFLU0085/DgcH) have a nucleotide identity of 71-84% and an amino acid identity of 68-92% (S1 Table). Thus, identical base pair substitutions are not always possible and, without studying each single case, the probability of parallelism at the nucleotide or amino acid level cannot be calculated and is not included in our predictions. Mutational hot spot sites with greatly increased mutation rates are also expected to contribute to parallelism when they are conserved but reduce parallelism when they are not. Based on analysis of patterns of mutations in SBW25 and *Pseudomonas aeruginosa* combined with homology modeling of protein structure using Phyre2 [16] (described in the supplementary methods section *Prediction of mutational targets and proposed molecular effects* below), regions expected to be mutated were predicted and the likely molecular consequences of different mutations suggested (Fig 2). The mutational effects on protein function can be directly connected to the mathematical model of the genotype-to-phenotype map by linking their likely effects to increasing or decreasing reaction rates (S1 Text *Analysis of mutational effects on reaction rates* below).

With limited experimental data from one species, it is likely that heterogeneities in mutation rates means that only a fraction of possible mutants are observed after experimental evolution. This difficulty is particularly obvious when considering genes where deletions can cause WS, in that a very large number of deletions are theoretically possible, considering all possible start and stop sites, while only a few are typically observed and often repeatedly so.  A way forward might be to use bioinformatics methods for predicting mutational effects, especially for cases where disabling mutations in a protein or certain region of a protein is to be predicted. However, mutational robustness of different types of proteins vary extensively [15], which could prevent a straight-forward application of a single method. Bioinformatics methods that predict effects of mutations in a single also give quite different results. For example, SNAP2 [17] , which relies mainly on evolutionary conservation, predicts that 49% of amino acid substitutions in WspF would be deleterious, while only a small subset of these mutations is observed in experiments. Another tool,  Suspect [18], that  also takes into account the 3D structure of a homology model of WspF, correctly predicts large effects for mutations in the interior of the protein and close to the enzymatic active sites, but fails to predict known surface-exposed sites where mutations cause loss of function that are believed to be important for interactions with WspA and WspE [14]. This highlights the problem that even with high-quality experimental 3D structures it will remain a major challenge to predict the effects of mutations on transient interactions with other large biomolecules.

A more easily solvable problem is that bioinformatics tools predict the functional effects on the protein level, without considering the possibility of single mutations to produce specific amino acid changes. Thus, an additional step would be needed to consider the codons in the gene to decide for which amino acid substitutions prediction of mutational effects need to be made.

**Prediction 8: Mutations will not be evenly spread between predicted Wsp, Aws, Mws proteins, but found mainly in those where mutations have the highest beneficial fitness effects**.

Even if the model of the genotype-to-phenotype map can accurately predict the rates of different WS mutants for Pf-5, previous results show that some WS mutants have lower fitness and are rarely observed after experimental evolution [6,14]. Thus, to produce more accurate predictions there is a need to *a priori* predict the relative fitness of different WS mutants using previous experimental data, assuming these will be conserved between species, or by theoretical considerations. The distribution of fitness effects of advantageous mutations has mainly been explored theoretically by Gillespie [19–21] and Orr [22,23], but this work assumes that the wild type is well adapted, which is not the case here as the wild type is a poor colonizer of the air-liquid interface. Noting that the most common mutations observed in SBW25 are likely to disable intermolecular or interdomain interactions [14] we instead turn to the literature on the distribution of fitness effects of mutations that *reduce* function and are therefore typically deleterious. The distribution of fitness effects of random mutations have been found to be bimodal for a large number of genes with different functions, with one mode close to neutrality and one corresponding to a complete loss of a particular molecular function [15,24–27]. In cases where disabling mutations in a gene are advantageous, we expect a similar bimodal distribution but that the second mode is centered at higher fitness than the wild type. However, there is no reason to expect that the mode of disabling mutations in different genes to be centered at the same fitness, which means that the observed WS mutants will be biased towards the genes with the highest fitness modes rather than distributed equally among them. The overall conclusion is that even if there are many possible genetic pathways to an adaptive phenotype in experimental evolution, we predict that mutations will only be found in the subset of genes with modes at highest fitness. If the ranks of relative fitness of the modes are conserved between species this would greatly aid predictions, but if they are not limited experimental data will be needed to predict the high fitness genes.

**Analysis of mutational effects on reaction rates**

An important aspect of testing the model is also to determine the effects on protein function of the mutations, which requires an analysis of individual mutations. For example, mutations in WspA could produce WS types by either disabling mutations affecting *r_2_* or enabling changes in *r_4_*. The molecular effects of the mutations found here are unknown, but knowledge from SBW25 and *P. aeruginosa* and their positions in protein structure allowed limited analyses of likely functional effects. Inactivating mutations in the negative regulator WspF were predicted to be either indels or missense mutations in four specific regions causing disabling changes to *r_2_* and *r_6_* in the model. Mutations were found in two of the predicted regions, one in the vicinity to the methylesterase active site where mutations are predicted to cause disrupt the catalytic site and reduce *r_2_* and the other one directly disrupting the phosphorylation active site in the signal receiver domain thereby reducing *r_6_*. No mutations were found in the surface exposed regions hypothesized to be involved in interactions with WspA and WspE, which could be due to differences in function between SBW25 and Pf-5 or simply that they appear at lower frequency and would be detected if additional mutations were isolated. The sole mutation in WspE is, as predicted, located in the direct vicinity of the phosphorylation active site and is predicted to reduce *r_6_*. Mutations in AwsX were amino acid substitutions throughout the gene as well as in frame deletions inactivating the gene as predicted that is expected to produce their phenotypic effect by reducing *r_3_,* although *r_3_* would also be reduced if function is completely disabled. Mutations in AwsR and MwsR were also found in predicted regions, but no mutations were found in the small periplasmic region of AwsR, which is the most commonly targeted region in SBW25 where it is likely to reduce *r_3_*. Known mutational hot spot sites in *awsX*, *awsR* and *mwsR* in SBW25 [14] were not conserved in Pf-5 resulting in divergent spectra of mutations, while mutated regions and predicted functional effects remain conserved between the two species. Little is known about the molecular function of the putative DGC encoded by PFL_0087/PFLU0085, but it is clear that a multitude of amino acid substitutions, deletions and insertions in a more than 40 amino acids long region can lead to WS [6]. Thus it functions as a small intragenic negative regulator region that have been proposed to be involved in oligomerization [28] and loss of this interaction results in constitutive activation of c-di-GMP production.

**Supplementary methods**

**Isolation of mutants with divergent colony morphology after experimental evolution**

After 5 days of experimental evolution in deep well plates, a 1 μl plastic loop was used to sample the populations by scraping at the bottom and at the wall and surface at the air-liquid interface. The cells on the loop were then transferred to an eppendorf tube with LB media and after repeated twists with the loop to disperse cells, the tubes were vortexed. Dilutions were plated on TSBGM plates with Congo Red and incubated at 36 °C for 48 h before being screened for divergent colony morphology. This procedure means that the sample taken does not represent a random sampling of the populations and therefore the frequency of divergent mutants is not an accurate measurement of the frequency of that mutant in the whole population. Neither can this frequency be directly compared to previous frequencies in SBW25. Divergent colonies typically represent from 0 to 20% of the colonies observed, but failure to see divergent colonies does not exclude that they are present at lower frequencies (<1%) in the populations. It is also possible that other adaptive mutants are present at significant frequencies in these populations, including mutants with increased ability to colonize the air-liquid interface without a change in colony morphology. However, for an adaptive mutant to increase in frequency from a single cell (even in the extreme case of already being present in the inoculum of about 10^3^ cells) to a relatively high frequency in the population, comparable to that of the divergent colony mutants (>0.5%), would require a high selection coefficient of s > 0.08 per generation.

The high frequency of identical WspF V271G mutations could possibly be due to pre-existing mutations that occurred during overnight growth under shaken conditions before the static experimental evolution started. To investigate this possibility we grew 30 replicates, each started with a single colony of the wild type Pf-5 in LB. After overnight growth, suitable dilutions were plated on LB agar plates and incubated at 30°C, conditions where the colony morphology of WspF V271G is most distinct from the wild type. No divergent colonies were observed for the 30 cultures among >5000 colonies screened suggesting that this mutant is unlikely to arise at such high frequency that it would be present in in inoculum of multiple independent populations in the experimental evolution experiment. As a positive control, one culture was spiked with 3% of WspF V271G before plating and this plate was easily identified by a researcher unaware of the presence of a spiked plate in the experiment. We are also certain that a subset of the overnight cultures used for experimental evolution were not started with colonies of the WspF V271G mutant already present in the frozen stock as these mutants are easily recognizable on agar plates. More WspF V271G mutants were found in the second round (three for first round compared to eight for the second) suggesting that there might be batch effects influencing the results. A two-tailed Fisher exact test of the number of this mutant compared to others, did not however find this difference significant (p = 0.08), but the relatively few mutants here means that only very strong batch effects could be detected.

**Reconstruction of mutations**

Thirteen mutations representing all candidate genes found as well as PFL_0087 and WspA mutations were reconstructed in the wild type ancestral *P. protegens* Pf-5 to show that they are the cause of the adaptive phenotype and to be able to assay their fitness effects without the risk of secondary mutations that might have occurred during experimental evolution. A two-step allelic replacement protocol was using to transfer the mutation into the ancestor. First a 1-2 kb fragment surrounding the putative adaptive mutations were amplified using PCR (Phusion High- Fidelity DNA polymerase, Thermo Scientific) and ligated into the multiple cloning site of the mobilizable pK18mobsac suicide plasmid (FJ437239) using standard molecular techniques. The ligation mix was then transformed into competent *E. coli* DH5α using heat shock. After confirmation of correct insert size by PCR the plasmid was transferred to *P. protegens* Pf-5 by conjugation with the donor strain and an *E. coli* strain carrying the conjugation helper plasmid pRK2013. Cultures were grown overnight of the recipient *P. protegens* Pf-5 (20 ml per conjugation at 30**°**C in LB) and 2 ml each of the donor and helper *E. coli* strains per conjugation at 37**°**C in LB with kanamycin. The culture of *P. protegens* Pf-5 was heat shocked for 10 minutes at 42**°**C prior to centrifugation at 4000 rpm for 10 minutes and resuspension in a small volume of LB. Donor and helper cells were collected by centrifugation 4000 rpm for 10 minutes, resuspended in LB, and mixed with the concentrated recipient cells. After another round of centrifugation the conjugation mix was resuspended in 50 μl LB and spread onto several spots on a LB agar plate followed by incubation overnight at 30**°**C. Each spot of the conjugation mix was scraped from the plate and resuspended in 200 μl LB each and plated on LB agar plates with kanamycin to select for transfer of the plasmid, and nitrofurantoin to prevent growth of the *E. coli* donor and helper cells. The pK18mobsac plasmid has a pBR322 type origin and cannot replicate in *P. protegens* Pf-5. Only cells where the plasmid has integrated into the chromosome by homologous recombination, with the homology provided by the cloned fragment, can grow in the presence of kanamycin. After streaking for single cells on LB agar plates with kanamycin, the *P. protegens* Pf-5 strains with integrated plasmids were grown overnight in LB at 30**°**C without antibiotics to allow for double crossover homologous recombination resulting in loss of the integrated plasmid. The plasmid also contains the *sacB* marker conferring sucrose sensitivity, which allows for counter-selection by plating on LB agar plates with sucrose. Sucrose resistant colonies were checked for loss of the kanamycin marker and DNA sequencing of the cloned region to find strains with the reconstructed mutation and no other mutations.

Deletion of the *wsp*, *aws*, *mws,* *pelABCDEFG* (PFL_2972-PFL_2978), *pgaABCD* (PFL_0161-PFL_0164) and *pslABCDEFGHIJKN* (PFL_4208-PFL4219) regions was accomplished using the same two-step allelic exchange protocol using SOE-PCR to generate a fragment surrounding the operon as previously described [1,2]. Gene synthesis (Thermo Fisher) was used to make DNA fragments used for deletion of PFL_0161-PFL_0164 and WspA T293-E299. Primer sequences are available in supplementary table S5.

**Fitness assays**

Two types of competition fitness assays were performed similarly to previously described assays [6]. The first assay measures invasion fitness, where a mutant is mixed 1:100 with the wild type ancestor, simulating early stages of air-liquid interface colonization where a rare mutant establishes and grows at the surface with no competition from other mutants. The second assay measures competition fitness in a 1:1 competition against a reference mutant strain. We chose the WspF V271G mutant because it was the most commonly found mutant during experimental evolution and thus is highly successful, either because of a high rate of emergence, *e.g.*, a mutational hot spot, or higher fitness than most other WS mutants. In addition, the WspF V271G mutant has a temperature sensitive colony morphology phenotype that it is highly wrinkly at 30**°**C but has a very mild phenotype when grown at room temperature, allowing it to be distinguishable from both the smooth ancestor and all other wrinkly mutants isolated here.

Fluorescent reference strains of the wild type ancestor and the WspF V271G mutants were created using a miniTn7 transposon (miniTn7(Gm) PA1/04/03 Gfp.AAV-a) [29] that allows integration at a defined locus (attTn7) in the chromosome. This allows the colonies to be distinguished not only by morphology, but by fluorescing under blue/UV light, and confers resistance to gentamicin. This provides a way to ascertain that secondary adaptive mutants that might occur during the competition experiment do not bias the results (for example the ancestor could evolve WS types or a WS mutant can evolve to cheat on the other type by inactivation of EPS production or reduced c-di-GMP signaling). Introduction of the transposon into *P. protegens* Pf-5 was performed by tri-parental conjugation from *E. coli* with helper plasmids pRK2013 (conjugation helper) and pUX-BF13 (containing the transposase genes) using the same conjugation protocol described above.

The invasion assay was performed by mixing shaken overnight cultures of the competitor 1:100 with the GFP-labeled reference ancestor followed by 1000-fold dilution and static incubation at 36**°**C for 48 h in TSBGM medium in deep well plates (1 ml per well, using only the central 60 wells). For the competition assay, the GFP-labeled reference strain WspF 271G was mixed 1:1 with the competitor and diluted 6-fold and grown for 4 h (shaken at 30**°**C), before plating to determine initial ratios, to ensure the cells were in a similar physiological state at the start of the competition. The competition cultures were then diluted 1000-fold in TSBGM medium and grown in deep well plates (1 ml per well, using only the central 60 wells) static for 24 h at 36**°**C. The entire population for each well was then transferred by carefully pipetting up and down repeatedly to gently disrupt the biofilm and collect cells that adhered to the walls of the well followed by vigorous vortexing in eppendorfs tubes. Selection coefficients (s) were calculated as previously described [30], where s = 0 is equal fitness, positive is increased fitness, and negative is decreased fitness relative to the reference strain. Briefly s is calculated as the change in logarithmic ratio over time according to s = [ln(R(t)/R(0))]/[t], where R is the ratio of mutant to reference and t is the number of generations of the entire population during the experiment (estimated from viable counts). The cost of the fluorescent marker were calculated from control competitions where the GFP-labeled reference strains (wild type and WspF V271G) were competed against isogenic strains without the marker and included in each plate under identical conditions during the fitness assays and used to adjust the selection coefficients to compensate for the cost. The competition and invasion fitness assays were designed to measure two different aspects of fitness likely to be relevant in the experimental evolution experiment based on previous work [6,2,14]. The competition assays measure the mutants’ ability to compete at the air-liquid interface with a highly successful mutant and the invasion assay measures the ability to colonize the air-liquid interface in the near absence of other adaptive mutants. Both these assays are conducted over shorter time scales (24 h and 48 h) compared to the experimental evolution experiment (5 days) to reduce the impact of secondary mutants arising during the experiment, which makes it impossible to assay mutants with low fitness. The final population sizes are similar in these assays and the experimental evolution experiment because the assays are started with a 1000-fold higher inoculum, which is important to reduce the effects of secondary mutants during the experiment.

Four replicates were used to measure competition fitness and six replicates for the invasion assay based on previous experience. A single colony of each competition strain was inoculated for overnight culture for each replicate and mixed with a reference strain. No outliers were excluded. If a large fraction of the colonies (>5%) display a phenotype caused by a secondary mutation during the fitness assay these would be excluded, for example if the wild type strain marked with GFP display a WS phenotype for the invasion assay. However, this did not occur for any of the replicates.

**Prediction of mutational targets and proposed molecular effects**

Predicted mutational targets in Fig. 2 is based mainly on regions and types of mutations, i.e. amino acid substitutions, and disabling in-frame or frame-shift insertions and deletions, previously found in *P. fluorescens* SBW25 [2,13,14,31,32] and *P. aeruginosa* [33–35]. This information was combined with the functional effects on reaction rates proposed in [14] and Uniprot annotations on functional sites:

Uniprot annotations:

*P aeruginosa* PAO1 *P. fluorescens* SBW25 *P. protegens* Pf-5

WspA: Q9HXT3 C3KE10 Q4KHM3

WspE: Q9HXT7 C3KE14 Q4KHL9

WspF: Q9HXT8 C3KE15 Q4KHL8

WspR: Q9HXT9 C3KE16 Q4KHL7

AwsX/YfiR: Q9I4L4 C3K218 Q4KIQ3

AwsR/YfiN: Q9I4L5 C3K217 Q4KIQ2

AwsO/YfiB: Q9I4L6 C3K216 Q4KIQ4

MwsR/MorA: Q9HVI8 C3K2D2 Q4K5S0

Structural homology models of the proteins listed above were made using Phyre2 (<http://www.sbg.bio.ic.ac.uk/phyre2>) [16] in intensive mode with the following program versions: Template detection: HHpred 1.51, Secondary structure prediction: Psi-pred 2.5, Disorder prediction: Disopred 2.4, Transmembrane prediction: Memsat_SVM, Multi-template modelling and *ab initio*: Poing 1.0

Quality and templates used by Phyre2:

WspA: 90% of residues modeled at >90% confidence, templates: 3zx6, 5jef, 1qu7

WspE: 88% of residues modeled at >90% confidence, templates: 1b3q, 1i5n, 2ayxA, c3i5a, 3bre, 2lp4, 2ch4, 3eq2, 4h60, 1w25

WspF: 100% of residues modeled at >90% confidence, template: 1a2o

WspR: 95% of residues modeled at >90% confidence, templates: 3bre, 3i5a

AwsO: 93% of residues modeled at >90% confidence, template: 4zhv, 4zhw

AwsR: 97% of residues modeled at >90% confidence, templates: 3ezu, 5jef, 4h54, c4zmuD, 1w25, 5llx

AwsX: 94% of residues modeled at >90% confidence, template: 4yn7

MwsR: 89% of residues modeled at >90% confidence, templates: 4rnh, 4hh2, 5akp, c5xgdA, 4f3l, 3lif, 4f3l, 3fos

To obtain protein coordinates for Pf-5, the amino acid sequences of the proteins from *P. aeruginosa* PAO1, *P. fluorescens* SBW25 and *P. protegens* Pf-5 was aligned using ClustalO (<https://www.ebi.ac.uk/Tools/msa/clustalo/>) [36].

Predicted mutated regions in Pf-5 were mapped onto the 3D homology models and expanded to include amino acid positions with likely similar effects on function, for example all amino acid residues surrounding an active site.

**Modelling of Wsp, Aws and Mws pathways**

The molecular networks of the three main pathways (Wsp, Aws and Mws) were modeled as a system of ordinary differential equations and this work is fully described as Model IV in [14] and a general description of the method is available in [37]. The model allows us to compute the relative likelihood that a mutation will translate into phenotypic change for the three main pathways to WS. Here we only use predictions from model IV and assume that mutations that produce disabling changes that reduce reaction rates are ten times more common than enabling changes. We also assume that the genotype-to-phenotype map is conserved and the modeled functions of the proteins in the Wsp, Aws and Mws are conserved, which means that there are no changes to the system of differential equations. This does not necessarily mean that all aspects of the molecular details of the systems are conserved and they can also, for example, respond to different environmental signals. Importantly, the model does not take into account the nature of the phenotype produced, in terms of exopolysaccharide used, as it computes the change to an indicator of phenotypic change, which is the active form of the DGC for each network. Therefore, results from the model can be directly used for prediction in Pf-5.

The first step is to describe the biochemical reactions of the molecular network by a set of ordinary differential equations (S1 Fig) that describe the reactions kinetics of the indicator of phenotypic change, in this case the active form of the DGC producing the c-di-GMP signal (R* for Wsp, RR for Aws and D* for Mws).

**S1 Fig** – Description of mathematical model of Wsp, Aws and Mws (adapted from [14]).

**S2 Fig** – Reaction networks of Wsp, Aws and Mws (adapted from [28]. The indicator of phenotypic change for each network (R* for Wsp, RR for Aws and D* for Mws) is shown in as bold edged circles.

To solve the system of differential equations knowledge of reaction rates and concentrations are needed. However, it is rarely possible to experimentally assay reaction rates and concentrations for proteins in regulatory networks. This modeling method was designed to produce useful predictions with limited knowledge of only of functional interaction network underlying a phenotype. To do so we choose uniform parameter distributions for initial concentrations U[0,10] and reaction rates 10^U[−2,2]^ for the interactions between components. Distributions for effects to reaction rates were chosen as 10^U[−2,0]^ for disabling changes and 10^U[0,2]^ for enabling changes.

The aim of model is to determine how the concentration of an indicator, in this case the active form of the DGC producing the c-di-GMP signal, change when reaction rates are changed up or down due to mutations. In order to link this to a phenotypic change we set a phenotypic threshold, compared to a previously determined baseline level, that here represents the wrinkly spreader phenotype. Numerical simulations were performed to repeatedly sample from the distributions of reaction rates, initial concentrations and magnitude of effect allows computation of the probability that each set of particular changes to reaction rates produce a wrinkly spreader.

The model also allows computation of the probability that changes to specific reaction rates in a network produce a wrinkly spreader. Changes to a reaction rate can be caused by mutational changes in all participating proteins and here we simply assume that mutations are equally likely in either one. An example of this for the Wsp network is to assume that disabling changes to *r_6_* occur with equal probability by mutations in either WspF or WspE. With knowledge of protein function this can be further modulated to include a predicted target size and an unequal distribution for the proteins in the interaction. However this did not improve predictions in [14], where equal numbers of mutations were found in WspA, WspE and WspF as predicted by the model, but where WspF is expected to have a larger target size.

**Construction of Multi-Locus Sequence Analysis phylogenetic tree**

The sequences of the four housekeeping genes 16sRNA, *gyrB*, *rpoB*, and *rpoD* were retrieved for 15 popular *Pseudomonas* species using available databases (GenBank accession numbers are listed in Supplementary Table S6). The sequences were subsequently concatenated in the following order: 16sRNA (1543 nt), *gyrB* (2430 nt), *rpoB* (4096 nt), and *rpoD* (1882 nt) [38] and a multi-locus alignment was created using the MEGA X software (version 10.1.7) [39] by implementing the MUSCLE algorithm. A phylogenetic tree was created using the maximum likelihood method and the Tamura-Nei model of nucleotide substitution [38].


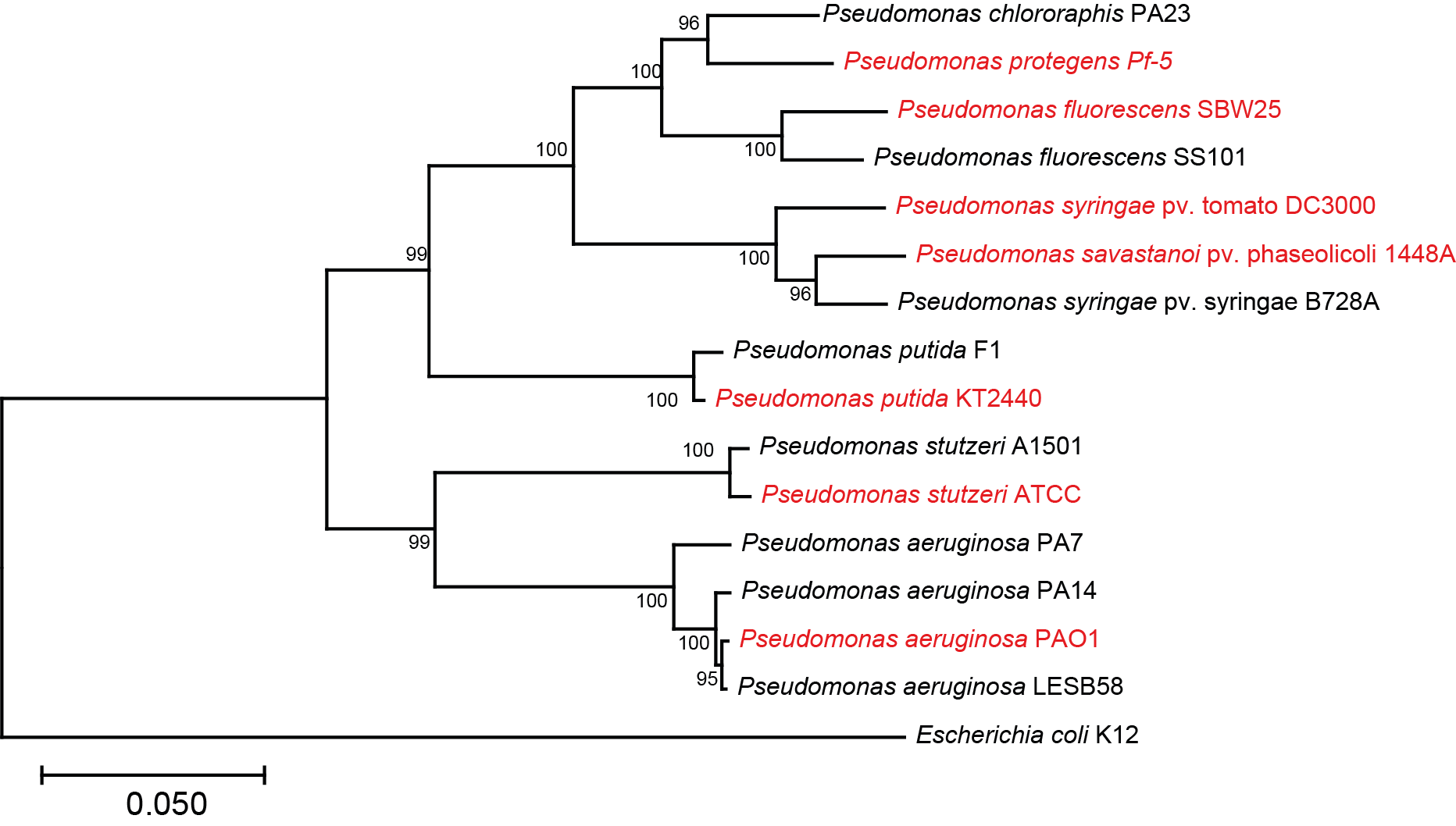


**S3 Fig** – Phylogeny of core genes for *Pseudomonas* species selected for evolutionary forecasting. Phylogenetic tree of 15 commonly studied *Pseudomonas* species based on the concatenated sequences of four genes (16sRNA, *gyrB*, *rpoB*, *rpoD*). Numbers at branch nodes indicate bootstrap values of 1000 replicates. The seven species used for further analysis of DGC and biofilm-related genetic diversity (Fig 7A, 7B) are labeled in red ((*P. fluorescens* SBW25, P*. protegens* Pf-5, *P. putida* KT2440, *P. syringae* pv. tomato DC3000, *P. savastanoi* pv. phaseolicola 1448A, *P. aeruginosa* PAO1, *P. stutzeri* ATCC 17588).

**References**

1. Ferguson GC, Bertels F, Rainey PB. Adaptive Divergence in Experimental Populations of Pseudomonas fluorescens. V. Insight into the Niche Specialist “Fuzzy Spreader” Compels Revision of the Model Pseudomonas Radiation. Genetics. 2013/10/01 ed. 2013. doi:10.1534/genetics.113.154948

2. Lind PA, Farr AD, Rainey PB. Evolutionary convergence in experimental Pseudomonas populations. ISME J. 2017;11: 589–600. doi:10.1038/ismej.2016.157

3. Beaumont HJ, Gallie J, Kost C, Ferguson GC, Rainey PB. Experimental evolution of bet hedging. Nature. 2009/11/06 ed. 2009;462: 90–3. doi:10.1038/nature08504

4. Gallie J, Libby E, Bertels F, Remigi P, Jendresen CB, Ferguson GC, et al. Bistability in a Metabolic Network Underpins the De Novo Evolution of Colony Switching in Pseudomonas fluorescens. Laub MT, editor. PLoS Biol. 2015;13: e1002109. doi:10.1371/journal.pbio.1002109

5. Friedman L, Kolter R. Genes involved in matrix formation in Pseudomonas aeruginosa PA14 biofilms. Mol Microbiol. 2004;51: 675–90. doi:10.1046/j.1365-2958.2003.03877.x

6. Lind PA, Farr AD, Rainey PB. Experimental evolution reveals hidden diversity in evolutionary pathways. eLife. 2015;4. doi:10.7554/eLife.07074

7. Wong A, Rodrigue N, Kassen R. Genomics of adaptation during experimental evolution of the opportunistic pathogen Pseudomonas aeruginosa. PLoS Genet. 2012;8: e1002928. doi:10.1371/journal.pgen.1002928

8. Römling U, Galperin MY, Gomelsky M. Cyclic di-GMP: the first 25 years of a universal bacterial second messenger. Microbiol Mol Biol Rev. 2013/03/09 ed. 2013;77: 1–52. doi:10.1128/MMBR.00043-12

9. Guttenplan SB, Kearns DB. Regulation of flagellar motility during biofilm formation. FEMS Microbiol Rev. 2013;37: 849–71. doi:10.1111/1574-6976.12018

10. Lee MC, Marx CJ. Repeated, selection-driven genome reduction of accessory genes in experimental populations. PLoS Genet. 2012/05/17 ed. 2012;8: e1002651. doi:10.1371/journal.pgen.1002651

11. Koskiniemi S, Sun S, Berg OG, Andersson DI. Selection-driven gene loss in bacteria. PLoS Genet. 2012;8: e1002787. doi:10.1371/journal.pgen.1002787

12. Hottes AK, Freddolino PL, Khare A, Donnell ZN, Liu JC, Tavazoie S. Bacterial adaptation through loss of function. PLoS Genet. 2013;9: e1003617. doi:10.1371/journal.pgen.1003617

13. McDonald MJ, Gehrig SM, Meintjes PL, Zhang XX, Rainey PB. Adaptive divergence in experimental populations of Pseudomonas fluorescens. IV. Genetic constraints guide evolutionary trajectories in a parallel adaptive radiation. Genetics. 2009/08/26 ed. 2009;183: 1041–53. doi:10.1534/genetics.109.107110

14. Lind PA, Libby E, Herzog J, Rainey PB. Predicting mutational routes to new adaptive phenotypes. Elife. 2019;8. doi:10.7554/eLife.38822

15. Kelley LA, Mezulis S, Yates CM, Wass MN, Sternberg MJ. The Phyre2 web portal for protein modeling, prediction and analysis. Nat Protoc. 2015;10: 845–58. doi:10.1038/nprot.2015.053

16. Gillespie JH. A simple stochastic gene substitution model. Theor Popul Biol. 1983;23: 202–15. doi:10.1016/0040-5809(83)90014-x

17. Gillespie JH. Molecular Evolution over the Mutational Landscape. Evolution. 1984;38: 1116–1129. doi:10.1111/j.1558-5646.1984.tb00380.x

18. Gillespie JH. The causes of molecular evolution. New York: Oxford University Press; 1991. Available: Publisher description http://www.loc.gov/catdir/enhancements/fy0638/91016709-d.html Table of contents only http://www.loc.gov/catdir/enhancements/fy0638/91016709-t.html

19. Orr HA. The distribution of fitness effects among beneficial mutations. Genetics. 2003;163: 1519–26.

20. Orr HA. The population genetics of beneficial mutations. Philos Trans R Soc Lond B Biol Sci. 2010;365: 1195–201. doi:10.1098/rstb.2009.0282

21. Firnberg E, Labonte JW, Gray JJ, Ostermeier M. A comprehensive, high-resolution map of a gene’s fitness landscape. Mol Biol Evol. 2014;31: 1581–1592. doi:10.1093/molbev/msu081

22. Jacquier H, Birgy A, Le Nagard H, Mechulam Y, Schmitt E, Glodt J, et al. Capturing the mutational landscape of the beta-lactamase TEM-1. Proc Natl Acad Sci U S A. 2013;110: 13067–72. doi:10.1073/pnas.1215206110

23. Jimenez JI, Xulvi-Brunet R, Campbell GW, Turk-MacLeod R, Chen IA. Comprehensive experimental fitness landscape and evolutionary network for small RNA. Proc Natl Acad Sci U S A. 2013;110: 14984–9. doi:10.1073/pnas.1307604110

24. Lind PA, Arvidsson L, Berg OG, Andersson DI. Variation in Mutational Robustness between Different Proteins and the Predictability of Fitness Effects. Mol Biol Evol. 2017;34: 408–418. doi:10.1093/molbev/msw239

25. Lundin E, Tang PC, Guy L, Nasvall J, Andersson DI. Experimental determination and prediction of the fitness effects of random point mutations in the biosynthetic enzyme HisA. Mol Biol Evol. 2017. doi:10.1093/molbev/msx325

26. Lambertsen L, Sternberg C, Molin S. Mini-Tn7 transposons for site-specific tagging of bacteria with fluorescent proteins. Environ Microbiol. 2004/06/10 ed. 2004;6: 726–32. doi:10.1111/j.1462-2920.2004.00605.x

27. Dykhuizen DE. Experimental studies of natural selection in bacteria. Annu Rev Ecol Syst. 1990;21: 373–398.

28. McDonald MJ, Cooper TF, Beaumont HJE, Rainey PB. The distribution of fitness effects of new beneficial mutations in Pseudomonas fluorescens. Biol Letters. 2011;7: 98–100. doi:10.1098/Rsbl.2010.0547

29. Goymer P, Kahn SG, Malone JG, Gehrig SM, Spiers AJ, Rainey PB. Adaptive divergence in experimental populations of Pseudomonas fluorescens. II. Role of the GGDEF regulator WspR in evolution and development of the wrinkly spreader phenotype. Genetics. 2006/04/21 ed. 2006;173: 515–26. doi:10.1534/genetics.106.055863

30. Malone JG, Jaeger T, Spangler C, Ritz D, Spang A, Arrieumerlou C, et al. YfiBNR mediates cyclic di-GMP dependent small colony variant formation and persistence in Pseudomonas aeruginosa. PLoS Pathog. 2010;6: e1000804. doi:10.1371/journal.ppat.1000804

31. Malone JG, Jaeger T, Manfredi P, Dötsch A, Blanka A, Bos R, et al. The YfiBNR signal transduction mechanism reveals novel targets for the evolution of persistent Pseudomonas aeruginosa in cystic fibrosis airways. PLoS Pathog. 2012;8: e1002760. doi:10.1371/journal.ppat.1002760

32. O’Connor JR, Kuwada NJ, Huangyutitham V, Wiggins PA, Harwood CS. Surface sensing and lateral subcellular localization of WspA, the receptor in a chemosensory-like system leading to c-di-GMP production. Mol Microbiol. 2012;86: 720–729. doi:10.1111/mmi.12013

33. Madeira F, Park YM, Lee J, Buso N, Gur T, Madhusoodanan N, et al. The EMBL-EBI search and sequence analysis tools APIs in 2019. Nucleic Acids Res. 2019;47: W636–W641. doi:10.1093/nar/gkz268

34. Libby E, Lind PA. Probabilistic Models for Predicting Mutational Routes to New Adaptive Phenotypes. Bio-protocol. 2019;9: e3407. doi:10.21769/BioProtoc.3407

35. Lind PA. Repeatability and predictability in experimental evolution. In: Pontarotti P, editor. Evolutionary biology. Cham: Springer; 2019. pp. 57–83. doi:10.1007/978-3-030-30363-1

36. Mulet M, Lalucat J, García-Valdés E. DNA sequence-based analysis of the Pseudomonas species. Environ Microbiol. 2010;12: 1513–1530. doi:10.1111/j.1462-2920.2010.02181.x

37. Kumar S, Stecher G, Li M, Knyaz C, Tamura K. MEGA X: Molecular Evolutionary Genetics Analysis across Computing Platforms. Mol Biol Evol. 2018;35: 1547–1549. doi:10.1093/molbev/msy09602/03/2021 14:03:00
