# Supplementary material for: Forecasting of phenotypic and genetic outcomes of experimental evolution in Pseudomonas protegens
Source: PLoS Genet. 2021 Aug 5;17(8):e1009722. doi: 10.1371/journal.pgen.1009722 (PMC8370652; doi:10.1371/journal.pgen.1009722)
Supplement: Attachment — Submitted filename: Response to Reviewer comments.docx [file pgen.1009722.s009.docx]

Dear Olivier,

We would like to thank you and the reviewers for very insightful and constructive comments that have clearly helped us improve the manuscript. Below we have addressed each of the comments from the reviewers point by point. In particular, we have shortened the manuscript by moving the detailed description of the rationale for our predictions to the S1 Text and more clearly described the advantages and limitations of an extended model system in the introduction, which were two of the main concerns of the reviewers. We have also clearly explained why not clones from all replicate populations were analyzed and explained the relevance of changing the experimental conditions further. We hope that the revised manuscript is now suitable for publication.

Yours sincerely

Jennifer Pentz and Peter Lind

**E1.** -The question tackled is complex and the reductionist approach used is relevant but need to be precisely defined and its powers and limits presented.

**Response:** We have added a new section in the introduction to better explain the strengths and weaknesses of the model system to the reader (lines 117-147):

“The WS model could be extendable to other Pseudomonas strains or species if it can be shown that mutations are not mainly strain-specific and the dominant selective pressure in terms of colonization of the air-liquid interface is dominant and robust to minor changes in environmental conditions. To put it simply, this means that closely related strains should, to some degree, evolve similar adaptive phenotypes by similar mutations in similar environments. An extended WS model would be ideal for testing evolutionary predictability because it is easy to isolate many clonal mutants using an initial step of screening for differences in colony morphology, characteristic of many mutants with increased ability to colonize the air-liquid interface in SBW25 [20,22,25]. This allows detection of mutants at <1% frequency in a population and reduces DNA sequencing costs. However, it also means that other types of adaptive mutants, including those adapting to other selective pressures, without divergent colony morphology are not included even if they reach high frequencies in the population. Strong selection allows short-term experiments where only one or two mutations are typically responsible for the adaptive air-liquid interface colonization phenotype and very few secondary mutations are typically present, which makes it straightforward to connect genotypes to phenotypes and fitness. The short timescale used, typically tens of generations, will however limit insights on the predictability of longer evolutionary trajectories.

Despite the reductionistic appeal of the WS system, there is considerable complexity both in the range of different phenotypic solutions to colonization of the air-liquid interface and in genetic pathways to the high fitness WS phenotype [23,20,21,31,22,26]. Moreover, this complexity would be even greater if it can be extended to other species as both DGCs and biofilm structural components, e.g. polysaccharides, are frequently gained by horizontal gene transfer and lost by deletions leaving a mosaic pattern of presence and absence that is not strictly linked to phylogeny. In many cases these genes are not essential, meaning that they can be deleted to allow finding alternative adaptive pathways [32,22]. Detailed genetic and phenotypic data on adaptive mutants from experimental evolution under static conditions is available almost exclusively from SBW25, which is currently a major limitation. If the WS system can be extended, it would be possible to iteratively improve models and update predictions based on new experimental data to be less dependent on the idiosyncrasies of an individual strain and environment combination.”

**E2.** -My understanding is that a real pedagogic effort was made to explain all the different levels of predictability, unfortunately I agree with reviewer 1 that it makes the paper way too long and finish having the opposite effect of making results less clear. This is not a review paper.

**Response:** We have moved the lengthy predictions to the S1 text (lines 2005-2218) and instead provided a brief summary with references in the main text results section (lines 165-215):

“**Predictions for experimental evolution of Pseudomonas protegens Pf-5**

Our predictions are based on previous experimental data from SBW25 and *P. aeruginosa* and theoretical considerations that are briefly presented here together with supporting references and explained in detail in the S1 text. We believe that forecasts of experimental evolution under novel conditions, such as for a new species and a new environment, need to start with identifying the dominant selective pressures that determines which phenotypes will have increased fitness before making genetic predictions. Each level will be dependent on correct higher-level predictions (except for prediction 4) meaning that, for example, if no mutants with increased ability to colonize the air-liquid interface evolve, we have no reason to expect that mutations would be found the predicted genes.

1. Mutants with increased ability to colonize the air-liquid interface by increased cell-cell or cell-wall adhesion will evolve and rise to high frequencies as these will be among the most fit classes of single-step adaptive mutants (Fig 1A) [33,21,22].

2. Mutants that use exopolysaccharides will have higher fitness that alternative phenotypic solutions with increased air-liquid interface colonization, such as lipopolysaccharides, capsular polysaccharides, cell-chaining or adhesive proteins (Fig 1B)[1–4]. As Pf-5 does not have genes required for biosynthesis of cellulose it is predicted to instead use Pel exopolysaccharide [34].
3. The most fit class of mutants with increased ability to colonize the air-liquid interface will have reduced motility due to activation of DGCs resulting in increased c-di-GMP and exopolysaccharide production, and selection against the cost of motility in biofilms (Fig 1D) [21,22,30,35–38].
4. Most mutations will cause loss of function, followed by promoter mutations and even less frequent activating mutations and double inactivating mutations (Fig 1C) [20,22,23,32,39].

5. Mutations in the molecular networks of the negatively regulated DGCs WspR, AwsR and MwsR will be most common route to WS due to a large mutational target size (Fig 1D) [21,23,26].
6. A previously developed mathematical model predicts that most mutations will be in WspA, WspE, WspF, AwsR, AwsX and MwsR [26] (Fig 1E). This prediction assumes that the functional interactions of the molecular networks are conserved between SBW25 and Pf-5 and that inactivating mutations are much more common than activating mutations. It does not assume that mutational hot spot sites are conserved between SBW25 and Pf-5.

7. Mutated regions for proteins in the Wsp, Aws and Mws pathways are predicted based on previous mutations in SBW25 [22,23,26,27,40] and *P. aeruginosa* [41–43] combined with analysis of homology models of protein structures [44] (Fig 2).

8. Mutations will not be evenly spread between predicted Wsp, Aws, Mws proteins with large mutational targets, but found mainly in those where mutations have the largest beneficial fitness effects [23,21,26]. This is because mutations with similar functional effects on a protein, for example a complete loss of function, will have similar fitness effects [45–49]. Based on the limited data from SBW25 we cannot predict which mutated proteins will produce the most fit mutants.

The outcome of experimental evolution will here likely be influenced by the combined effects of fitness of mutants and mutation rate to adaptive phenotypes, which is determined by mutational target size, i.e. number of sites, and mutation rate heterogeneities among these sites. The development of an improved, more principled, predictive model requires data from more than one strain as it will depend on the relative importance of these factors and to what extent they are conserved between different species or can be predicted a priori.”

We believe that the process used to make prediction and explaining how previous data was used and what assumptions were made is an important part of the article that would be of particular interest to researchers developing model systems for evolutionary forecasting. Yet we also understand the comments that this makes the article too long and difficult for the general reader to access, which is why we decided to move this part to the supplementary text instead of completely removing it. This is also in line with comment R3:4 from reviewer 3 that it needs to be clear how the predictions were made and what data was used.

**E3.** -all reviewers noticed the missing lineages and those should be clearly discussed.

**Response:** We have clarified (lines 266-269) the reason only mutants from 43 populations were characterized:

“For the remaining 17 wells, no mutants with clear changes in colony morphology was observed, but this does not exclude that such mutants could be present in the population at a lower frequency (<1%) or that other adaptive mutants were present”

We have also clarified the screening step in the introduction (lines 122-126) and expanded the description about how these mutants were isolated in the methods

section (lines 653-657)

**E4.** -Reviewer 3 has a strong point when he also mention the change of media. It could be turned into a benefice by noticing that despite these difference similarities were observed but it definitely needs to be addressed.

**Response:**
This is indeed a benefit and a key requirement for an extended WS system is that a dominant selective pressure can be established that not dependent on the specific experimental conditions as mentioned in the abstract (lines 22-25, 31-32) and discussed in the introduction (lines 84-87, 117-121), results section (lines 257-262), discussion (lines 505-509) and S1 text (lines 2025-2035). We have also clarified in the introduction what we mean with environmental robustness and dominant selection pressure (lines 117-121) to avoid this being perceived as arguing that environment will have no effect on fitness or which mutant will be the most fit. We simply want an experimental model system where the outcome is not strictly dependent on a very particular experimental set up, as this cannot be maintained between species with, for example, different optimal growth temperatures.

**Comments to the Authors:**

**Reviewer #1**: This manuscript addresses the predictability of short-term evolution across two species of Pseudomonas, one of which represents an iconic example of diversifying selection in a static environment with previously well described phenotypes and mutational hotspots. The authors ask here if similar phenotypes and similar mutations will evolve in another Pseudomonas species with a considerable divergence from the SBW25 strain previously studied. They also use different media and temperatures to test if the predictions from the previous workout strain adaptation were insensitive to these differences in the environment, as the dominant pressure should be access to oxygen. Furthermore, the authors also performed experimental evolution on selected deletion mutants of the most common pathways in the new species.
The manuscript brings new results on the short-term adaptation to this selective pressure by Pseudomonas Pf5, with a convincing set of well design experiments and an interesting choice of the new strain. It also provides a table of predictions on pathways that are expected to mutate in other species of pseudomonas when under selection to colonize the liquid air interface.

**Response:** Good summary of the article!

**R1:1.** However, I did find that the manuscript was sometimes unclear regarding what the new and the past results indeed were.

**Response:** We have removed the prediction and outcomes from Fig 1 which could be confusing to readers as it was not properly explained before. We also moved the lengthy prediction part of the results to supplementary text and instead provide a shorter summary with a list of predictions in the main text with references to the datasets used for predictions (lines 165-215) and where each prediction is further explained in the S1 text (lines 2005-2218).

**R1:2.** In general, I found the manuscript length a bit too long with many repetitions that appear unnecessary. Specially, when the repetitions refer to either hypothesis stated in other papers or models done in previous literature. Thus, the manuscript as currently written does not make rapidly clear to the reader what the novelty here really is.

**Response:** We have substantially shortened the manuscript by moving the long prediction part to the S1 Text (lines 2005-2218) to make it more clear to the reader what the novelty is while still allowing the reader to evaluate the basis for our predictions that will of course need to be dependent on previous work. We have also shortened the predictions for the other *Pseudomonas* species considerably (lines 445-470).

**R1:3.** For example, lines 126 to 143 are a bit repetitive with the introduction and do not appear to me as results. The same applies to lines 156-160.

**Response:** We have removed lines 126-143 and moved lines 156-160 to the supplementary text (lines 2013-2020) as an introduction to the long description of the predictions as we believe it would be of interest to understand the rationale for how our predictions were made and how this could be applied to other model systems for evolutionary forecasting.

**R1:4.** To me the results start on page 9 Line 190, where phenotypic predictions are made.

**Response:** We have moved this part to the S1 Text where it serves as a kind of introduction outlining the rationale for our prediction process (lines 2005-2020).

**R1:5.** The results of the genetic predictions could also be shortened, particularly lines 235- 259, and more directly go to the predictions from the modelling of reaction rates with a clear explanation on what is novel and what comes from previous work by the author(s).

**Response:** We have moved this part to the supplementary text (lines 2075-2099) and now simply state the predictions in the main text together with references to previous work (lines 188-193). We do however believe that lines 235-259 (now lines 2080-2099 in supplementary text) is useful as a more general prediction that could still apply when more detailed predictions on the gene level fails and we have linked this more clearly to a prediction why Wsp, Aws and Mws is expected to be the major mutational targets here.

Some minor specific comments are:

**R1:6.** Line 287- What is the rational for assuming a 10-fold difference here?

**Response:** We have described this further in lines 2125-2128 in the S1 text where this section has been moved to:

“Based on data from Lind et al. 2019 and the general expectation that disabling mutations (reducing reaction rates) are more likely than enabling mutations (increasing reaction rates), we assumed that disabling changes are ten times more common in line with results from SBW25 [14] and previous data on effects of random mutations [15] (Fig 1E).”

**R1:7.** Line 314 – What is meant by “Mutational hotspots with greatly increased mechanistic mutation rates”?

**Response:** We wanted to distinguish between the way mutational hot spots are sometimes used to describe a commonly mutated gene (usually one that has a large target size) and specific mutations that occur at increased rates, but this attempt clearly just made it more confusing. We have not changed this sentence to (lines 2155-2157 in the supplementary text):

“Mutational hot spots sites with greatly increased mutation rates are also expected to contribute to parallelism when they are conserved, but reduce parallelism when they are not.“

and changed to “hot spot sites” throughout the manuscript.

**R1:8.** Lines 418-421 It is not clear if this is a mutational hotspot due to high mutation rates of due to selection.

**Response:** That is absolutely right, and we have changed this to say (lines 311-313):

“Identical WspF V271G missense mutations accounted for 11 out of 15 mutations in this gene, which could indicate a mutational hot spot site or a greatly increased fitness relative to other WS mutants.”

We then comment on this in the discussion (lines 576-579):

“The repeated isolation of this mutant seems to be caused by a putative mutational hot spot site, given that it does not appear to have higher fitness than other WS mutants (Fig 5), but it is possible that its fitness could be higher under the specific conditions where it first evolved.”

**Reviewer #2:**

The authors present the results of an attempt to forecast phenotypic and genetic changes during a short-term adaptive radiation in static cultures of the bacterial genus Pseudomonas. The present study builds on extensive past work on the evolution of mutants of a particular strain of P. fluorescence colonizing the air-liquid interface (called wrinkly spreaders, WS), and use information about known target genes and phenotypic and fitness effects of mutations in this species to predict mutation targets and their frequencies in a strain of P. protegens after selection under related conditions. Their predictions are largely supported in this new species and under somewhat different conditions, while they also describe failures for some specific predictions, including the role of a novel polysaccharide to form WS mutants and the lack of contribution of mutations in WspA. They close by presenting predictions about the adaptive role of possible genes and pathways to yield WS mutants in a number of other Pseudomonas species based on genome content, but these are left untested.

**Response:** Good summary!

**R2:1**. Aspects we like about this study are the promising role of their experimental model for evolutionary forecasting events of adaptive radiation in other systems, and the careful consideration of the various aspects of the genotype-phenotype-fitness map that are relevant for their predictions, including a critical discussion of possible reasons for the failed predictions. Also, the experiments and analyses of evolved changes, including the construction and analyses of various mutants, are all sound and support the claims. What we feel is lacking, is a more fundamental, mechanistic model allowing such predictions, as many predictions now are direct extrapolations from previous observations in P. fluorescence, rather than predictions based on some underlying level of understanding of the effects and rates of the mutations involved. They do use a (previously published) metabolic model to predict relative contributions of the various WS mutations, and they do mention most likely mutation regions within target genes, and we consider these aspects as most valuable, as they indicate a more fundamental and generalizable way forward. Nevertheless, we think the study is suitable for publication in PLoS Genetics, as it provides a reasonable heuristic attempt to an ambitious goal using an interesting system for evolutionary forecasting of adaptive radiation events. We hope that our comments may help to improve readability and the reader’s appreciation of the implications of their work for the problem of evolutionary forecasting more generally.

**Response:**
We are glad that you consider this a promising experimental model for evolutionary forecasting, which is one of our main aims with this work, and thank you for your constructive comments. We would also like to have a more fundamental mechanistic model, but as data are now limited mainly to SBW25 it is clear that many predictions will be extrapolations (now mentioned in the introduction lines 142-144):

“Detailed genetic and phenotypic data on adaptive mutants from experimental evolution under static conditions is available almost exclusively from SBW25, which is currently a major limitation”

This does not mean predictions will necessarily be successful and we believe that additional data is required to develop more general models, which is our long-term goal with the larger project of using iterative cycles of prediction, experimental evolution and model updates to improve forecast for different *Pseudomonas* species (now described in the introduction lines 144-147:

“If the WS system can be extended, it would be possible to iteratively improve models and update predictions based on new experimental data to be less dependent on the idiosyncrasies of an individual strain and environment combination.”

and the discussion 618-627):

“Currently, given what is already known about the effects of unpredictable mutational biases and differences in fitness between different WS types, many of these forecasts will inevitably fail. However, hopefully they will fail in interesting ways, thereby revealing erroneous assumptions and providing possibilities for iterative improvement and development of more principled forecasting models. The ability to remove common genetic and phenotypic pathways provides a unique opportunity to also find those pathways that evolution does not commonly use. This is necessary to determine why forecasts fail and update the predictive models for another cycle of prediction, experimental evolution, and mutant characterization that make it possible to use this iterative model to define the information necessary to predict short-term evolutionary processes.”

There are many fundamental biological assumptions that would need to be made to develop a more general predictive model that takes into account the main determinants of evolutionary outcomes here (fitness, mutational targets size and mutation rate variation) and we hope that our work can contribute to developing such models although as outlined in the discussion we also see many challenges ahead.

Specific comments:
**R2:2.** It should be stated more clearly for which more general evolutionary questions the WS system is most suitable and promising in the context of evolutionary forecasting. Now, the findings presented are of interest to a rather narrow public of people working with the same system, while this model has clear potential to be relevant more broadly to questions relating to adaptive radiation, biofilm formation, etc. This is now mentioned only briefly at the end, but should be mentioned already in the introduction and discussed more thoroughly in the discussion.

**Response:**

We have added a section in the introduction (lines 117-147) to describe what we believe to be the benefits and disadvantages of this particular model system with regards to evolutionary forecasting. We disagree that the findings as now presented “are of interest to a rather narrow public of people working with the same system” in that much of our discussion concerns very fundamental questions about evolutionary predictability, including the predictability of fitness effects of mutations (lines 546-568)) and the challenges posed by variation in mutation rates across sites (lines 570-613) that are completely independent of the model system.

**R2:3.** The general appeal of the study would also benefit from a better presentation how the successful and failed predictions could be used to develop this system into a more principled predictive model, allowing predictions for other bacterial strains and conditions. For example, would it be possible to link the WS-producing networks of Fig. S2 to basic metabolism, e.g. along the lines recently done for streptomycin resistance in E. coli by Pinheiro et al. (2021, Nat Ecol Evol)? Also, the authors mention (lines 714-721) that mutation hotspots cannot be predicted from genome sequences, while they mention the known roles of repeat sequences and IS-elements. Could that information not be used to a first approximation to determine mutation target size and rates?

**Response:** Our long-term goal is to develop a more principled predictive model, but more data is needed to see to what degree genotype-to-phenotype maps, fitness effects of mutations and mutational hot spots are conserved. This will determine to what degree experimental data from another species can be directly used, what can be predicted *in silico* methods and what needs to be experimentally measured for each single species.

We have added a short comment about the possibility to use metabolic fitness landscapes and possible limitations in the discussion lines 561-568:

“An alternative way forward might be to develop metabolic fitness models [61] to predict the trade-off between the metabolic cost of producing exopolysaccharide and global effects on gene regulation from increased levels of c-di-GMP with the benefit derived from increased ability to colonize the air-liquid interface. But it is likely that such models would require extensive experimental data and if new data is required for each species or for every new environment more success might be achieved by measuring the fitness effect of a few targeted mutations in predicted genes to identify those with highest fitness (Prediction 8).”

With regards to predicting mutation rates and mutation target size we are actively pursuing these questions, but at this time it would be misleading to suggest that we are anywhere close to be able to do this in a way that allows more accurate forecasts. We have exemplified the problems with predicting mutations rates in lines 604-611:

“This difficulty can here be exemplified by attempting to predict hot spot sites in *awsX*, where an in-frame deletion between 6-bp direct repeats account for about 28% of the total mutation rate to WS in SBW25 [1], despite hundreds of possible WS mutations [1–4], while a 10-bp direct repeat in the same gene is used at a ten-fold lower rate. In Pf-5, only 5 bp of the 6-bp hotspot site in SBW25 is conserved, but another 8-bp direct repeat, where a deletion would cause an in-frame deletion, would be predicted to be used at a high rate; yet no such mutations are observed among the nine *awsX* mutants found here.”

It is a quite different problem to explain the possible mechanism behind a particular identified mutational hotspot compared to finding all hot spot sites in sequences of many thousands of base pairs, especially since many seemingly obvious hot spots are not seen experimentally and that some hot spots lack a clear mechanistic explanation.

A similar problem also applies to prediction of mutation target size where we only suggest regions likely to be mutated without specifying exact mutations. Currently we do not believe it is possible to accurately predict which mutations that would produce a WS phenotype, even for a protein like WspF where any inactivating mutation would give rise to a WS. There are several problems that are now discussed in S1 text lines 2166-2189:

“With limited experimental data from one species, it is likely that heterogeneities in mutation rates means that only a fraction of possible mutants are observed. This difficulty is particularly obvious when considering genes where deletions can cause WS, in that a very large number of deletions are possible, considering all start and stop points, while only few are observed and often repeatedly so. A way forward might be to use bioinformatics methods for predicting mutational effects, especially for cases where disabling mutations in a protein or certain region of a protein is to be predicted. However, mutational robustness of different types of proteins vary extensively [15], which could prevent a straight-forward application of a single method. Bioinformatics methods that predict effects of mutations in a single also give quite different results. For example, SNAP2[17] ,which relies mainly on evolutionary conservation, predicts that 49% of amino acid substitutions in WspF would be deleterious, while only a small fraction of these mutations is observed in experiments. Using another tool Suspect [18] that can also takes into account the 3D structure of a homology model of WspF, correctly predicts large effects for mutations in the interior of the protein and close to the active sites, but fails to predict known surface-exposed sites that are believed to be important for interactions with WspA and WspE [14]. This highlights the problem that even with high-quality experimental 3D structures it will remain a major challenge to predict the effects of mutations on transient interactions with other large molecules.

A more easily solvable problem is that bioinformatics tools predict the functional effects on the protein level, without considering the possibility of single mutations to produce specific amino acid changes. Thus, an additional step would be needed to consider the codons in the gene to decide for which amino acid substitutions prediction of mutational effects need to be made.”

**R2:4.** To aid readers not familiar with the WS system, a schematic overview of the genes and pathways involved in the biofilm formation at the surface of static growth tubes would be valuable addition. Maybe their current figure 1 could be incorporated in such an overview figure. Also, the purpose of Fig. 1 was not fully clear, particularly what prior information was used for the predictions, and that the outcome column summarizes the results of the present study rather than the success of previous predictions with the SBW25 strain. The predictions now seem to be based only on previous empirical observations in SBW25 and not to involve any fundamental reasoning about underlying causes and constraints.

**Response:**

Good idea! We have changed Fig 1D to include an overview of the function of Wsp, Aws and Mws and the connection to c-d-GMP, motility and EPS production.

To avoid confusion of what results were novel we have removed the prediction/outcome part of figure 1.

We have shortened the section on the predictions and what prior information was used based on comments from the editor. As we still believe it is critical for the reader to be able to assess the basis of the predictions, we have moved this section to the S1 text. It is an obvious limitation (mentioned in lines 142-144) that experimental data was only available from the SBW25 system and therefore many predictions are primarily based on empirical observations from this species. Indeed, a key motivation for this study was to obtain more data that might lead to more general predictive models (lines 144-147, 618-627).

However, we do not agree that predictions do “not to involve any fundamental reasoning about underlying causes and constraints”. For example, the prediction that mutants colonizing the air-liquid interface will evolve is based on the basic assumption that aerobic bacteria will grow a lot faster with access to oxygen that is available at the surface so it will be the dominant selective pressure. The prediction that most mutations will be loss-of-function mutations, followed by promoter mutations and then activating mutations may be inspired by the SBW25 system, but it is a very general prediction based on mutational targets size and higher mutation rates at promoters. The mathematical model of the Wsp, Aws, Mws molecular networks are purely theoretical based only on the functional connections of the proteins and how mutations can change the output in terms of activation of a DGC. While the model was tested with the SBW25 system no empirical data was used for the prediction here, except for a general assumption that disabling mutations are more common than enabling mutations. We **do** have detailed measurements of the mutation rate to WS in the absence of selection for the different pathways in SBW25 (Lind et al. eLife 2019) but this was not used here because of our assumption that mutational hot spot sites are not likely to be conserved.

**R2:5.** 17 out of the 60 Pf-5 lines did not colonize the air-liquid interface. How does this compare to SBW25 and what implication does this have for forecasting the outcome? Might this indicate that this biofilm formation is more constrained in Pf-5? Do these 17 lines represent alternative -rare but more fit- strategies to deal with oxygen depletion?

**Response:** We have added comments on the 17 wells from which no mutants were isolated (lines 266-269):

“For the remaining 17 wells no mutants with clear changes in colony morphology was observed, but this does not exclude that such mutants could be present in the population at a lower frequency (<1%) or that other adaptive mutants were present (see S1 text for extended discussion).”

And discuss in the S1 Text (lines 2251-2287):

**“Isolation of mutants with divergent colony morphology after experimental evolution**

After 5 days of experimental evolution in deep well plates, a 1 ml plastic loop was used to sample the populations by scraping at the bottom and at the wall and surface at the air-liquid interface. The cells on the loop were then transferred to an eppendorf tube with LB media and after repeated twists with the loop to disperse cells, the tubes were vortexed. Dilutions were plated on TSBGM plates with Congo Red and incubated at 36 °C for 48 h before being screened for divergent colony morphology. This procedure means that the sample taken does not represent a random sampling of the populations and therefore the frequency of divergent mutants is not an accurate measurement of the frequency of that mutant in the whole population. Neither can this frequency be directly compared to previous frequencies in SBW25. Divergent colonies typically represent from 0 to 20% of the colonies observed, but failure to see divergent colonies does not exclude that they are present at lower frequencies (<1%) in the populations. It is also possible that other adaptive mutants are present at significant frequencies in these populations, including mutants with increased ability to colonize the air-liquid interface without a change in colony morphology. However, for an adaptive mutant to increase in frequency from a single cell (even in the extreme case of already being present in the inoculum of about 10^3^ cells) to a relatively high frequency in the population, comparable to that of the divergent colony mutants (>0.5%), would require a high selection coefficient of s > 0.08 per generation.”

The absence of a high frequency (about 1%) of mutant with divergent colony morphology does not exclude that such mutants are present in the population at lower frequencies. It is also possible that biofilm mutants (or other adaptive mutants) without a divergent colony morphology could sometimes be present at significant frequencies that would prevent for example WS types to rise to high enough frequencies when they appear later. However, as these mutants are more difficult to isolate it would require vast amounts genome sequencing data and genetic reconstructions to even determine if they exist and are adaptive. It is not possible to directly compare frequencies or fitness measurements with SBW25 to fully elucidate the causes of differences because of for example differences in populations size and other experimental conditions and this was not a goal for this project.

**R2:6.** Line 217-219: Unclear why Pel is expected to be the primary EPS in Pf-5, and not one of the other two EPSs mentioned. It should be explained why this EPS is predicted to be preferred over CPS, LPS and PG.

**Response:** We have added the following section to the S1 Text describing the basis for the predictions (lines 2048-2059):

“In SBW25, at least four alternative distinct phenotypes with divergent colony morphology are selected for at the surface, involving an alternative EPS, CPS, LPS and PG, but they all have lower fitness than the WS type that form cellulose-based mats [1–4]. Pf-5 lacks genes for cellulose biosynthesis, but there are other EPSs that might be used (e.g., Pel, Psl, polyglucosamine (PGA)). Thus, we predict that overexpression of an EPS will always provide the most stable mats and therefore have the highest fitness (Fig 1C) and that Pel will be the primary EPS used based on its importance for pellicle formation at the air-liquid interface in Pseudomonas aeruginosa PA14 [5], the lack of mutants using Psl in and the lower fitness of mutants using PGA in SBW25 [2]”

**R2:7.** Line 303 and further: information about the nature of the different mutations, i.e. whether inactivating or more subtly altering the gene, would be helpful for evaluating the relative mutational targets. For example, how do the authors explain the remarkable repeatability of missense mutation V271G in WspF (line 418), and the absence of nonsense or frameshift mutations? What is their evidence that this is a mutational hotspot, as the authors believe? Is this not a gene that is inactivated by adaptive mutations? Perhaps predictions of these mutation target sizes, at least for SNPs, could be added to Fig. 2?

**Response:** We have added information about the proposed molecular effects of each mutation in S2 Table. The V271G mutation is believed to disrupt the demethylase site and while we suggest this is a mutational hot spot based on that it does not appear more fit than other WS, mutants we also allow for the opportunity that this mutant is more fit and therefore is found at higher frequency (line 311-313) and discussed in lines 576-579. Indeed, this is a gene that will give rise to WS when inactivated (as predicted in Fig 2) and nonsense and frameshift mutations have been found in SBW25. We cannot say if there are additional constraints in Pf-5 or that some mutants with complete loss of function would be less fit.

We do not believe that we currently can make more detailed predictions about target size in a way that would improve predictions, even for SNPs, as discussed above (R2:3) in in the S1 text (lines 2166-2189). We also note that addition of a simple type of mutational target size, such as gene size for negative regulators, would not improve forecasts in previous work in SBW25 (Lind et al. eLife 2019) where mutations in *awsX* has a higher mutation rate to WS than *wspF* despite being half as long. Another example is the similar mutation rates of *wspF* and *wspE* in SBW25 even though all mutations in WspE are clustered in a relatively small region, while any mutation inactivating WspF should produce a WS.

**R2:8.** Information about the total number of mutations found in the 43 evolved clones is lacking. The text mentions “secondary mutations”, but not how many. This may also clarify why mutants with single mutations were constructed and analyzed.

**Response:** We have added this information to S2 Table. Many of the WS mutations were found with Sanger sequencing (line 665-669) and in those cases, it is not known if there are secondary mutations, which is one reason why mutations were reconstructed before characterization.

**R2:9.** Line 456: what was tested in the ANOVA presented? It seems to support the statement that the second phenotype was less wrinkly and had similar motility, but unclear how.

**Response:** Thanks for pointing this out. Indeed, an ANOVA was used here to support the statement that the second phenotype (including the putative AwsXRO promoter capture and mutations in the promoter region of PFL_3078-3093) did not differ significantly from the WT motility using *posthoc* pair-wise comparisons. But, we agree that this was unclear and may not be necessary to support this statement. As such, we have removed the ANOVA. Additionally, the difference in phenotypes are clear from both the colony morphology and motility measurements combined.

**R2:10**. The section “Predictions for other Pseudomonas species” (p25-27) I found rather superfluous without presenting tests of those predictions. I would prefer a synthesis section summarizing (perhaps with a figure) the possible elements of an improved predictive model of WS evolution, but this might also be done in the discussion (see my 1st comment).

**Response:** We have shortened this section substantially (lines 445-470) but we still believe that making clear testable predictions for several *Pseudomonas* species, in addition to the more general steps and challenges for more predictive models described in the discussion, is a key part of the article and something that is rarely attempted. This is a clear application showing why it is important to find model systems where forecasts can be made for other species and immediately tested. We hope that an improved predictive model of WS evolution will eventually be possible by integrating experimental data from more *Pseudomonas* species, but that many fundamental difficulties remain to be addressed.

**Reviewer #3**: In this manuscript, the authors set out to determine if they could expand upon a well-characterized experimental system to predict the phenotypic and genotypic evolution of closely-related bacterial species. This manuscript addresses an important question in evolutionary biology – to what extent is evolution repeatable and thus predictable? Few studies have investigated, at a molecular level, how closely-related bacterial species adapt to a common selective pressure.

The authors find that the genetic routes of adaptation are mostly conserved between two species of Pseudomonas (despite differences in evolution conditions) and identify several routes that are specific to Pf-5 or SBW25. Despite the absence of the primary structural component of SBW25 mats (cellulose), Pf-5 adapts via mutations in the same c-di-GMP/DGC regulatory pathways. The predictions are extended to additional Pseudomonas species, which the authors intend to test in a future study.


MAJOR CONCERNS:

**R3:1.** I have several major concerns with the study. First, the study introduced two major variables, genotype and environment, yet the effect of each individual variable on evolutionary outcomes was not thoroughly investigated. The authors assumed that since the same genes were primary targets of selection in both the classical system and the extended system, that any differences in evolutionary outcomes are attributed to genotype rather of environment. I disagree with this assumption.

**Response:** We do not agree that the manuscript present this as that we assumed “that any differences in evolutionary outcomes are attributed to genotype rather of environment”. We have clarified what we mean (lines 117-121)

“The WS model could be extendable to other Pseudomonas strains or species if it can be shown that mutations are not mainly strain-specific and the main selective pressure in terms of colonization of the air-liquid interface is dominant and robust to minor changes in environmental conditions. To put it simply, this means that closely related strains should, to some degree, evolve similar adaptive phenotypes by similar mutations in similar environments.”

and in the abstract (lines 31-32):

“The experimental conditions were also changed compared to the SBW25 system to test if forecasts were extendable to a non-identical environment.”

Our goal was not to investigate “the effect of each individual variable on evolutionary outcomes” but rather to test if the WS system could be extendable to other species under slightly different environmental conditions (lines 22-25). We have also clarified this in the abstract (lines 31-32): “The experimental conditions were changed compared to the SBW25 system to test if forecasts were extendable to a non-identical environment.” This would require that related species, with similar gene content, evolve in somewhat similar ways to be able to use previous data and models (lines 117-121). It would also require that evolutionary outcomes are not dependent on a very particular environment (lines 117-121), simply because the influence of the environment will be very different depending on species, for example using the same temperature even when optimal growth temperatures are different. For forecasting to be successful for novel situations (and also for potential future applications), it must be possible to say something about how related strains evolve in similar environments rather than identical strains in identical environments.

**R3:2.** The authors’ claim of a dominant selective pressure suggests that the shape of the tail of the DFE (and the beneficial mutations that comprise it) remains intact despite the changes between the classical and extended systems. However, due to the substantial environmental changes, there must be a point at which the DFEs diverge, and it is unclear if this point was reached. I would need to see evidence that SBW25 adapts via the same genetic mechanisms in both the classical and extended system (especially beyond Wsp/Mws/Aws) before I’m convinced that other environmental components did not influence adaptation.

**Response:** We do not claim that other environmental components do not influence adaptation. It is clear though that in both cases adaptive mutants with similar phenotypes and similar mutations rise to high frequencies in the populations. We do not make any assumptions about the shape of the DFE and as we discuss in the S1text (lines 2189-2205) we do not think that theory about the shape of the DFE will be useful for forecasting in this particular case. We simply assume that one of the fittest classes of mutants are those that have increased ability to colonize the air-liquid interface because access to oxygen is one of the major factors limiting growth. This is of course not expected to be true under all environmental conditions. A simple example would be that we do not predict *P. syringae* to evolve such phenotypes under the conditions used here because it cannot grow at this temperature and therefore heat adaptation would be the dominant selective pressure. That SBW25 would adapt in exactly the same way under these conditions is not something we assume, and this question is much more limited in scope than what we are addressing here.

**R3:3.** Further, I think it is dangerous to rely on the assumption of a single selective pressure when extending predictions to additional species because any divergence in adaptation would be attributed to genetic background. I would be particularly concerned about species that are already well-adapted to colonize the air-liquid interface – thus shifting selection to other environmental components.

**Response:** We have clarified in the introduction that mutants adapting to other selective pressures are possible but that they are less likely to be isolated here as they might not have a divergent colony morphology (lines 122-128):

"An extended WS model would be ideal for testing evolutionary predictability because it is easy to isolate many clonal mutants using an initial step of screening for differences in colony morphology, characteristic of many mutants with increased ability to colonize the air-liquid interface in SBW25 [20,22,25]. This allows detection of mutants at <1% frequency in a population and reduces DNA sequencing costs. However, it also means that other types of adaptive mutants, including those adaptive to other selective pressures, without divergent colony morphology is not included even if they reach high frequencies in the population”.

Indeed, in cases where a species is already well adapted to colonize the air-liquid interface in a certain environment this prediction is likely to fail as we mentioned in the text (now in S1 text lines 2027-2030):

“Mutants with an increased ability to colonize the air-liquid interface will be one of the fittest class of adaptive mutants and rapidly increase in frequency due to the large increase in growth rate under high oxygen conditions (Fig 1A). This is likely to be true only when the wild type is a poor colonizer of the air-liquid interface.”

We do not believe that our predictions will be universally successful, and we do not consider failed predictions as failures as they will be an important part to producing better models and revealing erroneous biological assumptions.

**R3:4**. A second major concern is that, apart from gene targets, the predictions are too subjective. The predictions should be data-driven, yet some predictions are made without any connection to a data set. For example, there are no citations and no references to data to support the prediction that “mutants that specialize in colonizing the air-liquid interface will have the highest fitness” (lines 190-201). At times, the authors seem to pick and choose what data are used to generate predictions (e.g. that Pel with be the primary EPS based on its importance for pellicle formation at the air-liquid interface in PA 14, lines 218-219). I feel strongly that the authors need to specify the exact data set used to make their predictions.

**Response:** We have moved the lengthy description of predictions to the S1 text (lines 2005-2218) and now instead present this as a short list with supporting references in the main text (lines 176-208). We have added an extended explanation for the prediction of Pel in the S1 text lines 2051-2057:

“Pf-5 lacks genes for cellulose biosynthesis, but there are other EPSs that might be used (e.g., Pel, Psl, polyglucosamine (PGA)). Thus, we predict that overexpression of an EPS will always provide the most stable mats and therefore have the highest fitness (Fig 1C) and that Pel will be the primary EPS used based on its importance for pellicle formation at the air-liquid interface in Pseudomonas aeruginosa PA14 [5], the lack of mutants using Psl in SBW25 and the lower fitness of mutants using PGA in SBW25 [2].”

We agree that the prediction that “mutants that specialize in colonizing the air-liquid interface will have the highest fitness” is unclear and we have changed all mentions of “highest fitness” to “among the fittest classes of single mutation adaptive mutants”. The assumption that aerobic bacteria will grow faster with access to oxygen does not seem like a subjective prediction to us. In some cases predictions are based mainly on limited empirical observations in SBW25 and our interpretation of these data is sometimes more subjective and probably biased due to idiosyncrasies of the SBW25 system (discussed as a limitation in the introduction lines 142-144) and our solution is to try to develop an extended model system where “it would be possible to iteratively improve models and update predictions based on new experimental data to be less dependent on the idiosyncrasies of an individual strain and environment combination” (lines 145-147) . Predictions that are completely “data-driven” can only be used for identical situations and any extension would require subjective evaluations about the usefulness of the data for the novel situation.

**R3:5.** Finally, I believe that the manuscript would be greatly improved with heavy revisions. Specifically, much of the text lacks clarity. At times, the writing is too vague to convey meaning (e.g. “different results” line 81) and other times where descriptions need to be more concise (e.g. all predictions in the Results). As currently written, the reader needs to expel too much time/effort to follow the paper’s progression and authors’ logic.

**Response:** We have clarified line 81 to specifically say (lines 79-84): “I. Adaptive mutations are often strain specific, so that adaptation of different strains of the same species to an environmental challenge, such as high temperature, often result in mutations in different genes. For example, after adaptation to high temperature in minimal media with glucose for the *E. coli* strains K-12 MG1655 [11] and REL1206 [6], a *E. coli* B derivative, none of the top ten most frequently mutated genes were shared between strains. We have moved the long descriptions about the predictions to the S1l text and instead presented the reader with a list of predictions with references to reduce the time/effort that the general reader would need to understand the paper.

MINOR CONCERNS
**R3:6.** • It is unclear from the abstract if the focus of the study is the impact of the environment or genotype (or both).

**Response:** The focus of the study is the question “to what degree evolutionary forecasts can be extended to related species in non-identical environments” (abstract lines 22-25). It is not mainly about the impact of environment or genotype other than in sense that successful forecasting in an extended system would require that similar strains evolve in similar ways in similar environments. We have also clarified this in the abstract (lines 31-32): “The experimental conditions were changed compared to the SBW25 system to test if forecasts were extendable to a non-identical environment.”

**R3:7.** • In the Introduction (line 80), the authors state that “adaptive mutations are often strain specific” but the cited papers do not support this claim.

**Response:**

This was unclear and we have now clarified what we mean here (lines 79-84): “Adaptive mutations are often strain specific, so that adaptation of different strains of the same species to an environmental challenge, such as high temperature, often result in mutations in different genes. For example, after adaptation to high temperature in minimal media with glucose for the *E. coli* strains K-12 MG1655 [11] and REL1206 [6], a *E. coli* B derivative, none of the top ten most frequently mutated genes were shared between strains.”

**R3:8**. • The authors did not explain the necessity of the environmental changes. While I presume that some changes were necessary for growth of Pf-5, the abstract states the change was made “to test the robustness of forecasts to environmental variation”. The authors should explain why each of the environmental components were changed in the extended system.

**Response:** This is first discussed in lines 22-25 and we have further explained why it is important that the outcomes are not sensitive to the exact environmental conditions (lines 117-121). We have also changed the abstract to (lines 31-32):

“The experimental conditions were also changed compared to the SBW25 system to test if forecasts were extendable to a non-identical environment.”

The importance is that we can change the growth environment in many ways and still get similar results, which suggests it will work well as an extended model system for evolutionary forecasting. We are not focused on the impact of changes to a particular environmental component nor was there a particular scientific motivation for all changes to the experimental setup. For example, the switch from glass tubes to deep well plates was mainly because of it being more practical while use of media without animal components is preferred by some people for ethical or religious reasons.

**R3:9.** • The authors analyzed 43 clones isolated from 60 total populations. Why weren’t clones analyzed from the other 17 populations?

**Response:** We have clarified (lines 262-269):

“Air-liquid interface colonization was observed for the majority of the wells and independent mutants with clearly visible changes in colony morphology were isolated from the populations in 43 wells. One colony for each well was selected for further characterization at random based on a pre-determined position on the agar plate. For the remaining 17 wells no mutants with clear changes in colony morphology was observed, but this does not exclude that such mutants could be present in the population at a lower frequency (<1%) or that other adaptive mutants were present (see S1 text for extended discussion).”

**R3:10.** • Were non-divergent phenotypes considered as adaptive? How can mutants that colonize the air-liquid interface be most fit if no other mutants were analyzed? A minor point, but is it fair to say that the observed mutants are most fit since their fitness is density dependent? At high frequencies, they are likely less fit than other competitors.

**Response:** Good points. We have changed so we do not refer to these as most fit, but rather as “one of the fittest classes of single-step mutants”. It is of course impossible to prove that the fittest mutant has been found without assaying all possible mutants.

We have now clarified the screening for colony morphology in the introduction (lines 122-133). It is likely that fitness is frequency-dependent and this is mentioned as one of the limitations of the model system (line 515-518) in that it complicates fitness measurements and predictions of fitness.

**R3:11.** • This paper that compares the strategies of P. aeruginosa strains PAO1 and PA14 needs to be included in the discussion (DOI: 10.1128/mBio.02644-19)

**Response:** Thank you for the tip. We have added this as an example that there might be major differences even within the same species of *Pseudomonas*: Lines 540-544:

“It is also clear that strains of the same species can initiate biofilm formation in very different ways, as demonstrated for *Pseudomonas aeruginosa* PAO1 and PA14 [58], and that such differences would be difficult to predict without experimental data, which could potentially have a major impact on the predictability of evolutionary outcomes in an extended WS system.”

**R3:12**. • Are the entire evolution cultures plated to recover divergent phenotypes or just the mat? Same for the fitness assays. How is the mat disintegrated?

**Response:** Thank you for pointing out this omission. We have added more information about this in the methods section lines 653-657:

“A 1 μl plastic loop was used to collect cells from the bottom and the edges and surface of the air-liquid interface, that were then transferred to an Eppendorf tube with LB and vortexed vigorously. This sampling means that the frequency of mutants found is not necessarily representative for the entire population.”

This is also further explained in the S1 text lines 2252-2287:

“After 5 days of experimental evolution in deep well plates a 1 ml plastic loop was used to sample the populations by scraping at the bottom and at the wall and surface at the air-liquid interface. The cells on the loop was then transferred to an eppendorfs tube with LB media and after repeated twists with the loop to disperse cells, the tubes were vortex. Dilutions were plated on TSBGM plates and incubated at 36 °C for 48 h before being screened for divergent colony morphology. This procedure means that the sample taken does not represent a random sampling of the populations and therefore the frequency of divergent mutants is not an accurate measurement of the frequency of that mutant in the whole population. Neither can this frequency be directly compared to previous frequencies in SBW25. Divergent colonies typically represent from 0 to 20% of the colonies observed, but failure to see divergent colonies does not exclude that they are present at lower frequencies (<1%) in the populations. It is also possible that other adaptive mutants are present at significant frequencies in these populations, including mutants with increased ability to colonize the air-liquid interface without a change in colony morphology. However, for an adaptive mutant to increase in frequency from a single cell, even in the extreme cases of already present in the inoculum of about 103 cells, to a relatively high frequency in the population, comparable to that of the divergent colony mutants (>0.5%), would require a very high selection coefficient of s > 0.08 per generation.”

and

S1 text lines 2363-2365:

“The entire population for each well was then transferred by carefully pipetting up and down repeatedly to gently disrupt the biofilm and collect cells that adhered to the walls of the well followed by vigorous vortexing in eppendorfs tubes.”

**R3:13.** • It is my understanding that the mutants described here were chosen because of their divergent phenotype. There is no data presented in the manuscript that divergent phenotypes are more fit (or better colonizers of the air-liquid interface) than non-divergent phenotypes. The predictions in Fig 1A and Fig 1B were never actually tested. At the very least, the proportion of divergent colonies observed in each population should be included.

**Response:** We show with the invasion assays that the reconstructed mutants outcompete the ancestor strain. It is not straightforward to isolate other types of adaptive mutants from a predominantly wild type population where even the highly adaptive WS types are sometimes found at <1% (lines 122-133). This would require population genome sequencing at >>100X coverage followed by reconstructions of a large number of mutants, or genome sequencing of hundreds of clones, none of which we can afford, and we do not think this is relevant for this work.

Clearly prediction 1, that mutants with increased ability to colonize-the air-liquid interface would evolve was tested and such mutants were found suggesting that they are among the fittest classes of single step mutants. For prediction 2, we also show that the WS types that are partially dependent on Pel are more fit than those using the alternative polysaccharide (Pap). We think it is useful to make predictions, such that the most fit mutants will use EPS, without being able to fully test all other possibilities and prove that this is the truly case, but rather confirm that indeed such phenotypes evolve and are fitter than others found. It is also a prediction that can clearly fail, for example by finding an LPS mutant that outcompetes mutants that use EPS. The proportion of divergent colonies samples on the agar plates are not necessarily representative of the entire population because only a small part of the population is sampled (lines 655-657).

**R3:14**. • It’s unclear if the whole paper about access to the WS phenotype, divergent phenotypes, colonizers of the air-liquid interface, or whatever mutants are most fit.

**Response:** We have rewritten and expanded the introduction (line 117-147) to better explain how screening for divergent colony morphology (like the WS) allows isolation of mutants with increased ability to colonize the air-liquid interface. We have also clarified that this does not exclude that other adaptive mutants are present in the population (line 126-128, 266-269).

**R3:15.** • There is no explanation for the claims of “mutational hotspots”. What evidence exists that these loci experience an increased mutation rate? Have you ruled out the possibility that the mutations observed >1x were present in the inoculum – especially WspF V271G?

**Response:** It is extremely unlikely that these were present in the inoculum and this is further explained in the S1 text lines 2270-2287:

“The high frequency of identical WspF V271G mutations could possibly be due to pre-existing mutations that occurred during overnight growth under shaken conditions before the static experimental evolution started. To investigate this possibility we grew 30 replicates, each started with a single colony, of the smooth wild type Pf-5 in LB. After overnight growth suitable dilutions were plated on LB agar plates and incubated at 30°C, conditions were the colony morphology of WspF V271G is most distinct from the wild type. No divergent colonies were observed for the 30 cultures among >5000 colonies screened suggesting that this mutant is unlikely to arise at such high frequency that it would be present in in inoculum of multiple independent populations in the experimental evolution experiment. As a positive control one culture was spiked with 3% of WspF before plating and this plate was easily identified by a researcher unaware of the presence of a spiked plate in the experiment. We are also certain that some of the overnight cultures used for experimental evolution were not started with colonies of the WspF V271G mutant already present in the frozen stock as these mutants are easily recognizable on agar plates. More WspF V271G mutants were found in the second round (three for first round compared to eight for the second) suggesting that there might be batch effects influencing the results. A two-tailed Fisher exact test of the number of this mutant compared to others, did not however find this difference significant (p = 0.08), but the relatively few mutants here means that only very strong batch effects could be detected.”

We have also changed all mentions of mutational hot spot in Pf-5 to “putative mutational hot spot site” and explained that even though we did not detect any fitness differences compared to other WS mutants it is possible that the V271G mutant have a selective advantage: lines 311-313:

“Identical WspF V271G missense mutations accounted for 11 out of 15 mutations in this gene, which could indicate a putative mutational hot spot site or a greatly increased fitness relative to other WS mutants.”

and lines 575-579:

“the high frequency of Wsp mutants is mainly due to repeated V271G mutants. The repeated isolation of this mutant seems to be caused by a putative mutational hot spot site, given that it does not appear to have higher fitness than other WS mutants (Fig 5), but it is possible that its fitness could be higher under the specific conditions where it first evolved.”

We do not find this explanation implausible based on previously reported mutational hot spot sites in SBW25 where mutants were isolated without selection for air-liquid interface colonization (Lind et al. eLife 2019).

**R3:16.** • The model predicts an abundance of mutations in the Wsp pathway and this was observed. Most of the Wsp mutations were identical (WspF V271G), which the authors infer to be a mutational hotspot. However, the authors state that their model doesn’t take hotspots into account (line 284), so this outcome doesn’t actually support the model.

**R3:17.** • Since hotspots were not taken into account in the model, wouldn’t the presence of hot spots skew the

**Response:** The model predicts that most mutations would be found in the Wsp pathway, so the outcome does support the model in a basic sense. As we cannot predict mutational hot spot sites from sequence data (discussed in lines 601-613) and we do not assume that mutation hot spots sites are conserved between species, we could not include this in the model by increasing the probabilities of enabling and disabling mutations. This is mentioned in the S1 Text (lines 2121-2124):

“The model can also incorporate information about mutational biases by increasing probability of changes in specific reaction rates, which improved the predictive ability of the model in Lind et al. 2019 [14]. We do not assume that mutational hot spot sites are conserved and therefore all reaction rates had the same probability of mutational changes”

The distribution of mutation rates is largely unknown (as discussed in lines 601-613). It is likely to be broad with major variation in mutation rate but the frequency of sites with greatly increased mutation rates is unclear. Thus, even if V271G is the most obvious candidate for a mutational hot spot site, identical mutations are also found multiple times in AwsX and MwsR which could mean that these also have higher than average mutation rates. It is possible that for genes with many different adaptive mutations the average mutation rate to an adaptive phenotype might be similar despite variations in mutation rates between sites, which could mean that heterogeneities in mutation rates might have a limited impact on the order of genes mutated in cases where they have a large mutational target size.

**R3:18.** • Throughout the paper, wording needs to be changed to reflect that polysaccharides aren’t encoded by genes (their synthases are).

**Response:** Thank you for pointing out our mistake. We have changed this in all instances.

**R3:19.** • I fundamentally disagree with the message in lines 154-168 that evolutionary predictions need to follow a specific progression. I would argue that genotypic and phenotypic predictions can be made independent of one another. Regardless, I don’t think this paragraph needs to exist in the paper. It’s difficult to follow the progression of the paper because the predictions and results follow different order.

**Response:** We have moved this section together with the lengthy description of predictions to the S1 text. We do still want to keep it there to explain our way of thinking, which seems relevant given the fundamental disagreement expressed by the reviewer. We have replaced it in the main text with a shorter rationale for our predictions (lines 166-174):

“Our predictions are based on previous experimental data from SBW25 and P. aeruginosa and theoretical considerations that are briefly presented here together with supporting references and explained in detail in the S1 text. We believe that forecasts of experimental evolution under novel conditions, such as for a new species and a new environment, need to start with identifying the dominant selective pressures that determines which phenotypes will have increased fitness before making genetic predictions. Each level will be dependent on correct higher-level predictions (except for prediction 4) meaning that, for example, if no mutants with increased ability to colonize the air-liquid interface evolve, we have no reason to expect that mutations would be found the predicted genes.”

We do not here discuss “evolutionary prediction” in general but “forecasting experimental evolution in a novel context” (now S1 text lines 2013-2014). There might be some very general genetic predictions that can be made independent of phenotypes, such as those made in Prediction 4 (lines 188-190), or that genetic predictions in terms of mutational patterns during mutation accumulation might be possible. We fail to see how specific genetic prediction, e.g. mutations in a particular gene, can be made without a connection to what phenotypes will be adaptive in a certain environment. It is of course possible to make genetic predictions based on simple repeatability, or using fitness models made from large datasets (as discussed in lines 491-502), but this requires the implicit assumption that the novel situation is in some important way similar to the one where the previous data was gathered from. In particular cases, where adaptive mutations compensate for the fitness costs of known deleterious mutations, it might be possible to predict mutation targets without explicitly considering which phenotypes could be adaptive (for example *pyrE*-*rph* mutations in *E. coli* MG1655). We would be interested in successful evolutionary forecasts where genotypic and phenotypic predictions are made independently and hope to see such research published soon.

**R3:20. •** I don’t think the role of c-di-GMP and DGCs (and their impact on matrix formation) are explained well enough for a general audience.

**Response:** We have added a short description to the introduction in lines 108-110:

” The second messenger c-di-GMP is a highly conserved signal for biofilm formation in bacteria that controls formation of a matrix composed of polysaccharides, DNA and proteins, and reduce motility [29,30].”

**R3:21.** • Were there any batch effects attributed to the two independent evolution experiments?

**Response:** We have added batch number to mutants in S1 Table . The V271G mutation is found more commonly in round 2 (8 vs. 3 in round 1), but this is not a significance difference due to the limited number of mutants (S1 Text, lines 2283-2287).

**R3:22.** • The methodology used to predict the location of mutations was not explained (lines 316-318).

**Response:** We have added a major section with detailed description of how the predictions were made in the S1 Text methods sections (lines 2395-2437):

“Predicted mutational targets in Fig. 2 is based mainly on regions and types of mutations, i.e. amino acid substitutions, and disabling in-frame or frame-shift insertions and deletions, previously found in *P. fluorescens* SBW25 [2,13,14,31,32] and *P. aeruginosa* [33–35]. This information was combined with the functional effects on reaction rates proposed in [14] and Uniprot annotations on functional sites:

Uniprot annotations:

*P. aeruginosa* PAO1	*P. fluorescens* SBW25	*P. protegens* Pf-5

WspA: Q9HXT3	C3KE10	Q4KHM3

WspE: Q9HXT7	C3KE14	Q4KHL9

WspF: Q9HXT8	C3KE15	Q4KHL8

WspR: Q9HXT9	C3KE16	Q4KHL7

AwsX/YfiR: Q9I4L4	C3K218	Q4KIQ3

AwsR/YfiN: Q9I4L5	C3K217	Q4KIQ2

AwsO/YfiB: Q9I4L6	C3K216	Q4KIQ4

MwsR/MorA: Q9HVI8	C3K2D2	Q4K5S0

Structural homology models of the proteins listed above were made using Phyre2 (http://www.sbg.bio.ic.ac.uk/phyre2) [16] in intensive mode with the following program versions: Template detection: HHpred 1.51, Secondary structure prediction: Psi-pred 2.5, Disorder prediction: Disopred 2.4, Transmembrane prediction: Memsat_SVM, Multi-template modelling and ab initio: Poing 1.0

Quality and templates used by Phyre2:

WspA: 90% of residues modeled at >90% confidence, templates: 3zx6, 5jef, 1qu7

WspE: 88% of residues modeled at >90% confidence, templates: 1b3q, 1i5n, 2ayxA, c3i5a, 3bre, 2lp4, 2ch4, 3eq2, 4h60, 1w25

WspF: 100% of residues modeled at >90% confidence, template: 1a2o

WspR: 95% of residues modeled at >90% confidence, templates: 3bre, 3i5a

AwsO: 93% of residues modeled at >90% confidence, template: 4zhv, 4zhw

AwsR: 97% of residues modeled at >90% confidence, templates: 3ezu, 5jef, 4h54, c4zmuD, 1w25, 5llx

AwsX: 94% of residues modeled at >90% confidence, template: 4yn7

MwsR: 89% of residues modeled at >90% confidence, templates: 4rnh, 4hh2, 5akp, c5xgdA, 4f3l, 3lif, 4f3l, 3fos

To obtain protein coordinates for Pf-5, the amino acid sequences of the proteins from *P. aeruginosa* PAO1, *P. fluorescens* SBW25 and *P. protegens* Pf-5 was aligned using ClustalO (https://www.ebi.ac.uk/Tools/msa/clustalo/) [36].

Predicted mutated regions in Pf-5 were mapped onto the 3D homology models and expanded to include amino acid positions with likely similar effects on function, for example all amino acid residues surrounding an active site.”

**R3:23.** • The experiment is not designed such that the most prevalent mutant is necessarily the most fit. Early mutations that allow occupation of the A-L interface have a distinct advantage over late occurring mutations. Therefore, the prevalence of a mutant may be more of a consequence of when the mutation occurred than the magnitude of its fitness. Pathways with a large target size (or increased mutation rate) would be over-represented.

**Response:** We agree with this interpretation and indeed it is a key element of the article and previous work that is referenced here throughout the article (Lind et al. eLife 2015 and Lind et al. ELife 2019, Lind et el. ISME J 2017). That is why we focus not only on fitness effects of mutations but also on the rate at which the phenotypes arise due to differences in mutational target size and mutational hot spot sites. Prediction of all three aspects is presented as key challenges and is extensively discussed in lines 210-215, lines 547-614, S1 text lines 2101-2217. We can also measure the fitness of rare mutants (like PFL_0087) and mutants that are expected but not observed (like WspA) (lines 316-325) to further explore if less common mutants have higher fitness or if their rarity can be explained by lower fitness. We also specifically comment on the importance of priority effects in SBW25 in lines (588-590) in that early low fitness mutants are observed early on but are later outcompeted by WS types.

**R3:24.** • I disagree with the conclusions drawn from Fig. 6A and 6B, specifically that “Pel production is activated by mutations leading to increased c-di-GMP” (lines 530-531). The data indicate that Pel contributes to the ability of the mutants to colonize the A-L interface but not that Pel production was impacted by the mutations. Direct evidence is needed to show that Pel production is increased in the mutants.

**Response:** Deleting Pel in the absence of WS mutations does not impact fitness under these conditions, which was previously written in the Fig 6 legend. To clarify this, we have now moved it to the main text (lines 414-416):

“Deletion of the pel operon in the absence of WS mutations did not significantly reduce fitness in invasion against the wild type (0.020 +/-0.050 (SD), two-tailed t-test p = 0.38) or in competition against the wild type (-0.011 +/-0.024 (SD) two-tailed t-test p = 0.49).”

Given the previous results showing that Pel production can be activated by mutations increasing c-di-GMP we think that our suggestion (lines 416-419):

“This suggests that Pel polysaccharide serves as an important structural component for colonizing the air-liquid interface and that Pel production is activated by mutations leading to increased c-di-GMP levels”

is very reasonable, but it would need to be experimentally confirmed to be certain.

**R3:25.** • Adjacent black bars makes it impossible to compare individual genes across species in Fig. 7.

**Response**: Thank you for pointing this out. We have changed the figure to allow better comparisons between individual genes across species.

**R3:26.** • Has the whole genome sequence data been deposited into a public database?

**Response:** We have deposited the data at NCBI under accession number BioProject PRJNA737653 and added this information to the methods section Lines 681-682. We also put the accession numbers for each strain into S2 Table.

**Have all data underlying the figures and results presented in the manuscript been provided?**
Large-scale datasets should be made available via a public repository as described in the *PLOS Genetics* [data availability policy](http://journals.plos.org/plosgenetics/s/data-availability), and numerical data that underlies graphs or summary statistics should be provided in spreadsheet form as supporting information.

Reviewer #1: Yes

Reviewer #2: Yes

Reviewer #3: **No:** Fig 4B, Fig 6B missing underlying data

**Response:** We have added the motility data as S7 Table.

PLOS authors have the option to publish the peer review history of their article ([what does this mean?](https://journals.plos.org/plosgenetics/s/editorial-and-peer-review-process#loc-peer-review-history)). If published, this will include your full peer review and any attached files.

If you choose “no”, your identity will remain anonymous but your review may still be made public.


**Do you want your identity to be public for this peer review?** For information about this choice, including consent withdrawal, please see our [Privacy Policy](https://www.plos.org/privacy-policy).

Reviewer #1: No

Reviewer #2: No

Reviewer #3: No
